# Discovery of Early-Branching *Wolbachia* Reveals Functional Enrichment on Horizontally Transferred Genes

**DOI:** 10.3389/fmicb.2022.867392

**Published:** 2022-04-25

**Authors:** Nicholas Weyandt, Shiva A. Aghdam, Amanda M. V. Brown

**Affiliations:** Department of Biological Sciences, Texas Tech University, Lubbock, TX, United States

**Keywords:** plant-parasitic nematode, *Wolbachia*, enrichment, phylogenomics, endosymbiont, metagenomics

## Abstract

*Wolbachia* is a widespread endosymbiont of insects and filarial nematodes that profoundly influences host biology. *Wolbachia* has also been reported in rhizosphere hosts, where its diversity and function remain poorly characterized. The discovery that plant-parasitic nematodes (PPNs) host *Wolbachia* strains with unknown roles is of interest evolutionarily, ecologically, and for agriculture as a potential target for developing new biological controls. The goal of this study was to screen communities for PPN endosymbionts and analyze genes and genomic patterns that might indicate their role. Genome assemblies revealed 1 out of 16 sampled sites had nematode communities hosting a *Wolbachia* strain, designated *w*Tex, that has highly diverged as one of the early supergroup L strains. Genome features, gene repertoires, and absence of known genes for cytoplasmic incompatibility, riboflavin, biotin, and other biosynthetic functions placed *w*Tex between mutualist C + D strains and reproductive parasite A + B strains. Functional terms enriched in group L included protoporphyrinogen IX, thiamine, lysine, fatty acid, and cellular amino acid biosynthesis, while dN/dS analysis suggested the strongest purifying selection on arginine and lysine metabolism, and vitamin B6, heme, and zinc ion binding, suggesting these as candidate roles in PPN *Wolbachia*. Higher dN/dS pathways between group L, *w*Pni from aphids, *w*Fol from springtails, and *w*CfeT from cat fleas suggested distinct functional changes characterizing these early *Wolbachia* host transitions. PPN *Wolbachia* had several putative horizontally transferred genes, including a lysine biosynthesis operon like that of the mitochondrial symbiont *Midichloria*, a spirochete-like thiamine synthesis operon shared only with *w*CfeT, an ATP/ADP carrier important in *Rickettsia*, and a eukaryote-like gene that may mediate plant systemic acquired resistance through the lysine-to-pipecolic acid system. The Discovery of group L-like variants from global rhizosphere databases suggests diverse PPN *Wolbachia* strains remain to be discovered. These findings support the hypothesis of plant-specialization as key to shaping early *Wolbachia* evolution and present new functional hypotheses, demonstrating promise for future genomics-based rhizosphere screens.

## Introduction

Host-associated microbes may play significant roles in the rhizosphere, but the complexity of these environments can make them challenging to study. Diverse microbiota has been detected in nematodes, whose communities number in the millions per m^2^ in the rhizosphere (Bongers and Ferris, 1999; Adam et al., 2014; Ferris and Tuomisto, 2015; Elhady et al., 2017; Zheng et al., 2020; Topalović and Vestergård, 2021). Such microbes may facilitate plant-feeding in rhizosphere organisms, as has been observed in above-ground plant-feeders, in which symbionts supplement essential nutrients (Moran et al., 2008; Bennett and Moran, 2013) or synthesize protective toxins or plant toxin-degrading enzymes (Cheng et al., 2013; Douglas, 2015; Gressel, 2018). Microbes could play significant roles in the biology of plant-parasitic nematodes (PPNs), which cause up to 25% of global crop yield loss and cost ∼$100 billion annually (Nicol et al., 2011). Recent findings suggest PPNs can harbor mutualist symbionts, like *Xiphinematobacter* (Verrucomicrobia) (Vandekerckhove et al., 2000; Brown et al., 2015) and *Xiphinematincola* (Burkholderiales) (Palomares-Rius et al., 2016, 2021), or symbionts with unknown phenotypes, including *Cardinium* (Bacteroidetes) (Noel and Atibalentja, 2006; Brown et al., 2018) and *Wolbachia* (Alphaproteobacteria) (Haegeman et al., 2009; Brown et al., 2016), but current studies on the diversity and roles of such microbes are limited.

Genus *Wolbachia* includes the most remarkable and widespread symbionts, which can act as obligate mutualists (Foster et al., 2005; Taylor et al., 2012; Nikoh et al., 2014) or parasites that manipulate host reproduction to promote transmission through the female germline using a wide array of phenotypes. Reproductive manipulating *Wolbachia* phenotypes include male-killing, parthenogenesis induction, feminization, or cytoplasmic incompatibility (CI), wherein infected males mating with uninfected females fail to produce progeny (Werren, 1997; Taylor and Hoerauf, 1999; Werren et al., 2008). Whereas *Wolbachia* is widespread in insects, its discovery in PPNs has significant implications, given the ecological importance of PPNs: if these *Wolbachia* are obligate mutualists like filarial nematode *Wolbachia*, then disruption of the symbiosis could reduce the PPN burden on plants, whereas if they are reproductive parasites conferring CI as in many insects, this phenotype could be harnessed for biocontrol analogous to *Wolbachia*-based control programs in mosquitoes (Caragata et al., 2016; Carey, 2018).

However, to date, the role of PPN-type *Wolbachia* remains ambiguous. For example, based on an analysis of the available genome derived from strain *w*Ppe from the root-lesion nematode, *Pratylenchus penetrans*, comparative genomics could not fully resolve *Wolbachia*’s role (Brown et al., 2016). Phylogenomic analyses placed them at the root of the *Wolbachia* clade, supporting the early emergence of *Wolbachia* in ecdysozoan plant-diet specialists, perhaps supporting this diet. However, the genomic analysis suggested *w*Ppe was devoid of most pathways typically seen in diet-supplementing *Wolbachia* such as riboflavin, biotin, thiamine (Brown et al., 2016). Alternatively, it may serve as a facultative nutritional mutualist synthesizing heme or mediating host iron homeostasis (Gill et al., 2014; Brown et al., 2016, 2018), or perhaps co-synthesizing fatty acids or methionine when in the presence of dual infection with *Cardinium* (Brown et al., 2018). Conversely, *Wolbachia*’s variable prevalence and correlation with female-biased populations in its PPN host *P. penetrans* (Wasala et al., 2019) hint at reproductive manipulation, while the high prevalence of its most closely related strain, *w*Rad in the burrowing nematode, *Radopholus similis* (Haegeman et al., 2009), hints at possible mutualism. Unfortunately, to date, only one *Wolbachia* genome has been sequenced from a PPN (Brown et al., 2016).

Despite this limited genomic data and just three strains confirmed in three hosts (Haegeman et al., 2009; Brown et al., 2016; Brown, 2018; Wasala et al., 2019), PPN *Wolbachia* may be widespread. The evidence that PPN *Wolbachia* may be widespread derives from the evidence of *Wolbachia*-like horizontal gene transfers both recently and deeper in the nematode phylogeny (McNulty et al., 2010; Dunning Hotopp, 2011; Koutsovoulos et al., 2014; Husnik and McCutcheon, 2018), suggesting ancestral *Wolbachia* symbiosis even in lineages without current evidence of *Wolbachia*. Adding to this argument, current surveys of *Wolbachia* distribution may include false negatives, due to low titer infections, or PCR-screens with primers having mismatches to PPN-type *Wolbachia* (supergroup L) (Bordenstein et al., 2003; Augustinos et al., 2011), or inadvertent clearing of the symbiont resulting from routine antibiotic during culturing or processing of PPNs.

Therefore, to broaden our understanding of the early-branching *Wolbachia* in PPNs, this study screened rhizosphere nematode communities for *Wolbachia*. We performed phylogenomics and comparative genomics from metagenomic assembled genomes (MAGs), which has previously proven successful to gain insights into unculturable taxa (Brown et al., 2016; Scholz et al., 2020). We took advantage of genome skimming approaches (Denver et al., 2016; Myers et al., 2021) to simultaneously characterize PPNs, and also screened public databases. Mirroring recent work (Bennett et al., 2021; Myers et al., 2021), we also analyzed functional enrichment and signatures of selection to investigate *Wolbachia*’s role and transitions in function during evolution. The outcomes supported previous hypotheses about *Wolbachia*’s origin in plant-feeding nematodes and generated new hypotheses about specific core metabolic functions and horizontally transferred genes for symbiont-mediated nutrient pathways such as heme, lysine, and thiamine.

## Materials and Methods

### Field Sample Collection and Nematode Isolation From Soil and Roots

As part of a field survey seeking to uncover new nematode-associated endosymbionts, approximately 100–500 g of soil and roots were collected using a soil auger or serrated shovel, collecting from the top 15 cm of soil at a 30 cm to 1 m distance from the base of various plants at different sites (Supplementary Table 1). All handling and processing of samples were performed in compliance with USDA APHIS permits to A.M.V.B. Samples were kept cool (<10°C) until processed to isolate nematodes as described previously (Myers et al., 2021). Briefly, soil and roots were placed in Baermann funnels for 3–5 days, allowing nematodes to be collected. Nematodes were further separated from soil by sucrose flotation following (Jenkins, 1964) to remove remaining soil particles, and finally further washed to remove further contaminants by mixing in 40 mL distilled water, centrifuging, and discarding the supernatant.

### DNA Extraction, PCR Pre-screening, and Illumina Library Preparation and Sequencing

To characterize nematode community and *Wolbachia*-like DNA, total nematode community DNA was isolated from each sample. After a brief examination under an inverted microscope, about 800 to 1,400 nematodes from each sample were exposed to five cycles of freeze-thaw to break cuticles. DNA was isolated using the Qiagen DNeasy Blood & Tissue Kit (Valencia, CA, United States) following the manufacturer’s directions. DNA quantity and quality were assessed on the Nanodrop spectrophotometer. An initial sample was prepared for sequencing and analysis without pre-screening as follows: approximately 0.6–1 μg of DNA was used for genomic library preparation with the QIAseq FX 96 DNA Library Kit (Valencia, CA, United States) following the manufacturer’s directions with fragmentation times and AMPure bead size selection steps optimized for 450–550 bp fragments. Library quality and quantity were assessed on the TapeStation 2200 (Agilent, USDA). Libraries were normalized and pooled before sequencing on Illumina HiSeq, with 150 PE cycles performed at Genewiz, Inc. (NJ). After positive results for a plant-parasitic nematode-associated *Wolbachia* strain were obtained from one sample (following bioinformatics analysis described below), subsequent samples from the same farm were pre-screened for the presence of *Wolbachia* prior to sequencing with custom primers designed based on published qPCR assay primers (Mee et al., 2015) that showed a high sensitivity for low-titer infections. However, as these primers had mismatches to 16S rRNA gene sequences from plant-parasitic nematode-associated *Wolbachia* strains, we modified the primer sequences to increase specificity to our targets using aligned sequences. The resulting new primers were: forward Wol-mee-F 5′-CTC ACA GAA AAA GTC CT-3′ and reverse Wol-mee-R 5′-CGC CTT TAC GCC CAA T-3′, with thermal cycle conditions: 95°C for 2 min, 35 cycles of 95°C for 30 s, 59°C for 30 s, 72°C for 1 min, and one cycle of 72°C for 10 min.

### Draft Genome Assembly

To recover nematode and symbiont genomes, reads were *de novo* assembled (see details in Supplementary Material), then *Wolbachia*-like contigs were annotated. First, reads for each sample were filtered and trimmed using Trimmomatic version 0.38 (Bolger et al., 2014), and overlaps in paired reads were detected and merged in Pear version 9.11 (Zhang et al., 2014). Filtered paired reads and merged reads were *de novo* assembled with metaSPAdes version 3.13 (Bankevich et al., 2012; Nurk et al., 2017) using low kmers (25, 33, and 45). Assembly quality was assessed using Quast version 5.0.1 (Gurevich et al., 2013). Assemblies were screened for *Wolbachia*-like 16S rRNA using a two-step analysis with blastn in Blast + version 2.10.1 (Camacho et al., 2009) (−evalue 10) first to a custom database of *Wolbachia* 16S rRNA sequences, and then a second blastn to the complete NCBI nt database. Any samples with top blastn hits to PPN *Wolbachia* from this read-based blast were also considered “positive.” For PPN *Wolbachia*-positive samples, full genomes were extracted using similar two-step blastn searches, first to *Wolbachia* genome databases, then to the full nt database.

Based on the high sequence similarity among samples, and their origin from the same farm, we combined these samples for further analysis, to improve coverage and assembly quality. We used an iterative map-assemble approach using bwa version 1.17 (Li and Durbin, 2009) and a subtractive mapping approach as follows. First, we identified non-*Wolbachia* contigs in the initial assembles using both blastn results and a%GC filter using prinseq-lite.pl in the BRAbB software (Brankovics et al., 2016), then we used bwa mem to map each sample’s reads to this non-*Wolbachia* data specific to our samples, then we used samtools version 1.9 (Li et al., 2009) and custom scripts to extract unmapped (i.e., *Wolbachia*-enriched) reads. These enriched reads were concatenated for all samples and assembled in metaSPAdes with a kmers (25, 45, 65, and 99). The resulting assemblies were combined with *Wolbachia*-like contigs from the individual samples with the new strain. The process of bwa-based subtractive mapping was repeated from the original reads with this new, improved database. The new *Wolbachia*-enriched reads were again concatenated and *de novo* assembled again with metaSPAdes. This approach was repeated three times and stopped once the sum of the length of the resulting new strain *Wolbachia*-like contigs ceased to increase between cycles and inspecting genome contamination and completeness metrics using CheckM v1.0.18 (Parks et al., 2015) at intermediate steps.

The resulting contigs were assessed by several quality controls to reduce the likelihood of spurious bioinformatic contamination with non-*Wolbachia* data. To filter out contigs with potential short horizontally transferred *Wolbachia*-like DNA regions (HGTs), long contigs (>5,000 bp) were removed if coverage was >2 times the average coverage of the longest contigs, using coverage analysis in the pileup.sh in BBMap version 38.9 (Bushnell, 2014). To filter out possible HGTs, contigs were imported into Geneious Prime version 2020.0.4 (Biomatters, Ltd.) and inspected, with contigs >1,000 bp removed if GC content was below 24% or above 42%. Quality was assessed by annotating contigs using Prokka version 1.14.6 (Seemann, 2014) which uses Prodigal for *ab initio* gene prediction, HMMER3 for protein family profiles, BLAST+ for comparative annotation, Barrnap^1^ for rRNAs, Aragorn (Laslett and Canback, 2004) for tRNAs. The resulting genes were then analyzed with blastn to the nt database and with DIAMOND blastx to the full nr database to keep only contigs with the highest similarity to PPN *Wolbachia*. Finally, to check for regions of possible bifurcating misassembly due to mutational differences in the field sampled specimens, contigs were aligned and checked for blocks of near identity and synteny using Geneious Prime plugins ProgressiveMauve v1.1.1 (Darling et al., 2010) and LASTZ alignment v7.0.2 (Biomatters, Ltd.).

### Gene and Pathway Annotation

The final assembly quality was analyzed in Quast version 5.0.1 (Gurevich et al., 2013) and re-annotated in Prokka. Completeness and contamination were assessed in CheckM and by evaluating the genome presence of housekeeping genes and tRNAs. Pathways were assessed by gene presence-absence comparisons with other *Wolbachia* and outgroups Anaplasmataceae using Roary version 3.13 (Page et al., 2015), and using ModelSEED version 2.6.1 (Seaver et al., 2021) which assesses metabolic models in the KEGG and MetaCyc databases. ModelSEED was run with “complete” supplemented *in silico* media.

### Nematode Community Analysis and Nematode-*Wolbachia* Abundance Correlation

To assess possible nematode hosts for the *Wolbachia* from our sampled nematode communities, we compared 21 sampled nematode communities (Supplementary Table 1) that were extracted and sequenced as described above, as part of a broader study. All partial cytochrome oxidase I (COI) sequences with top blastn match to nematodes were extracted from our scaffolds using blastn and custom scripts. The relative abundance of nematodes in each sampled community was then estimated based on kmer coverage with the equation *C* = (*C*_K_.*R*)/(*R* − *K* + 1), where *C* is total coverage, *C*_K_ is kmer coverage, *K* is the length of kmers, and *R* is read length and normalization to the total number of reads sequenced for each sample. To this matrix of COI hits, we added a column with the normalized coverage of *Wolbachia* 16S rRNA for the *Wolbachia*-positive samples. We then calculated and plotted Spearman rank correlation (rho) values and *p*-values using the R package Hmisc version 4.5-0 ‘rcorr’ which calculates a matrix of Spearman’s rho rank correlation coefficients for all pairs of columns for non-missing elements, using midranks for ties, with method “spearman,” order “hclust,” hclust.method “average” and plotting using ‘corrplot’. Multiple testing correction was performed using the Benjamini and Hochberg (1995) method in R with p.adjust “BH.”

### Sequence Read Archive Screening

To survey publicly available sequencing projects for potential PPN *Wolbachia*-like sequences, we developed scripts that take input queries such as geographic locality, sample type, sequencing strategy, and access the NCBI Sequence Read Archive (SRA) data, then analyze downloaded datasets to screen for *Wolbachia.* Keywords include “rhizosphere,” “soil,” “grassland,” “forest,” “agriculture,” “nematode,” and strategies/platforms such as “MiSeq,” “HiSeq,” “Illumina,” “Amplicon,” or “wgs,” and combinations of these terms. Briefly, these scripts use the E-utilities public API from NCBI to obtain and analyze serially tabular sets of raw read data for SRA projects *via* concatenated SRR, ERR, and DRR run data, subjecting them to raw read 16S rRNA gene two-step blastn, as described above, using for the custom blastn all PPN *Wolbachia*, including the new *Wolbachia* 16S rRNA gene from the present study. For SRA experiments with positive results, reads were further assessed after adapter and quality trimming and filtering using Trimmomatic and merging using Pear as described above. The resulting *Wolbachia*-like sequences were then analyzed using phylogenetic analysis approaches described below.

### Phylogenetic and Phylogenomic Analysis

To understand the evolutionary relationships among candidate PPN hosts, candidate PPN-type *Wolbachia* strains from SRA data mining, and the new *Wolbachia* strain compared to other strains, various phylogenetic analyses were performed. In most cases, the general approach involved first blastn searches of our sequences of interest to the nt database at NCBI to find ∼100–500 most closely related sequences. We then download and aligned these using either Mafft version 1.0.4 (Katoh and Standley, 2013) or Clustal Omega 1.2.3 (Sievers et al., 2011), and trimming and removing duplicates or highly similar sequences within the Geneious Prime version 2020.0.4 (Biomatters, Ltd.) suite, prior to phylogenetic analysis using both maximum likelihood (ML) phylogeny reconstruction was performed in RAxML version 4 (Stamatakis, 2014), assessing bootstrap support from 500 replicates and Bayesian phylogeny estimation with MrBayes version 2.2.4 (Huelsenbeck and Ronquist, 2001; Ronquist et al., 2012) with final phylogenetic trees visualized in FigTree version 1.4.4^2^ with labels and color added in Adobe Illustrator. Specific phylogenetic analysis approaches are as follows.

For inferring relationships among candidate PPN hosts predicted from community correlation analyses (described above), partial nematode cytochrome oxidase 1 (COI) genes that were significantly correlated with *Wolbachia*-positive samples were aligned and analyzed with the ML GTR Gamma nucleotide model, with rate heterogeneity alpha estimated, and with rapid bootstrapping and search for the best-scoring ML tree (-f a -x 1) and Bayesian analysis with the GTR + G model with 4 categories, and Markov chain Monte Carlo settings of chain length 1,100,000, 4 heated chains, heated chain temp 0.2, subsampling frequency 200, Burn-in length 100,000, with random seed 31,569, and priors with unconstrained branch lengths GammaDir (1,0.1,1,1), checking for convergence with minESS > 200.

For inferring relationships among candidate PPN-type *Wolbachia* strains from SRA data mining, an initial set of thousands of trimmed and merged reads with top blastn similarity to PPN-type *Wolbachia* were aligned to reference 16S rRNA genes, then identical sequences were removed. Preliminary phylogenetic analyses in FastTree v2.1.11 (Price et al., 2010) with the GTR model were used to generate a preliminary tree to remove large numbers of sequences from the alignment that displayed exceptionally long branches, similar to the distance separating *Ehrlichia*/*Anaplasma* and *Wolbachia* clades, on the basis that these sequences may reflect either non-*Wolbachia* alphaproteobacterial, or possibly degrading 16S rRNA pseudogenes or degrading horizontally transferred 16S rRNA fragments. The resulting subset of SRA sequences was analyzed in three separate alignments for the sub-region of the 16S rRNA gene in which they occur, and then together in a larger alignment to include all sequences together. RaxML and MrBayes phylogenies were reconstructed as described above.

For inferring relationships among the new *Wolbachia* strain and other *Wolbachia* strains, we focused on three sets of analyses: two which included strain *w*Rad which was the only other strain to date from a PPN, and one which included a larger number of genes even though *w*Rad could not be included. For strain *w*Rad, only 3 gene regions have been sequenced. The first analysis was able to include additional early-branching *Wolbachia* isolates for which only the 16S rRNA gene sequence was available, and this analysis included additional outgroups *Candidatus* Neowolbachia serbia and *Candidatus* Mesenet longicola (GenBank Accessions MH618374.1 and BNGT01000041.1, respectively) and related bacteria from *Harpalus pennsylvanicus*, as well as additional Rickettsiales and outgroup alphaproteobacterial. The second analysis included more gene regions available for strain *w*Rad, including 16S rRNA, partial CTP synthase and ftsZ, and partial groES and groEL (GenBank Accessions EU833482.1, EU833483.1 EU833484.1, respectively) and corresponding gene regions for other *Wolbachia* and outgroups with gene regions with blastn matches to these from the NCBI nt and genome databases. Once aligned, the sequence block was concatenated for strains with all regions covered, and the final alignment was stripped of sites with gaps or ambiguities. We produced additional reduced-length alignments from this full gap-stripped alignment to test for potential long-branch attraction artifacts. These reduced alignments were generated by successively stripping sites with the highest evolutionary rates identified using TIGER v2.0 (Cummins and McInerney, 2011). Phylogenetic analyses on resulting blocks were performed as described above.

For a more robust phylogenomic analysis of PPN-type *Wolbachia* with their outgroups, we prepared an alignment of a larger number of core genes from full *Wolbachia* and outgroup genomes downloaded from NCBI. We used Roary to generate a codon-based alignment (core_gene_alignment.aln) block for analysis. As for the previous analyses, to control for potential alignment artifacts, we removed all positions with ambiguities or gaps and performed TIGER analysis to create additional shorter alignments with high evolutionary rate sites progressively removed, to potentially reduce the effects of long-branch attraction. ML and Bayesian analyses were performed as described above for each alternative alignment. Finally, using the initial codon-based alignment, we performed *in silico* translation, removed gaps and ambiguities, and performed ML analysis of amino acid sequences was performed with RaxML using the PROTGAMMAGTR substitution model with empirical base frequencies and 500 bootstrap replicates.

### Gene Repertoire Comparisons *via* Gene Ontology Enrichment Analysis

To evaluate gene repertoire differences among *Wolbachia* strains and outgroups and analyze functional gene ontology (GO) enrichment, we first performed gene annotation on our new *Wolbachia* strain assembly as well as 200 other draft Anaplasmataceae genomes from NCBI using Prokka with identical parameters for all assemblies. We then created ortholog sets using Roary v3.13.0 (Page et al., 2015) which performed blastp on gff files from Prokka, with parameters -e for codon-aware alignment in PRANK (Löytynoja, 2014) and -i 60 to allow for detection of distant orthologs with outgroups. Initial Roary results suggested some draft genomes were potentially incomplete (too few genes) or contaminated (too many genes), so this genome set was reduced to the best genomes, comprising 93 draft genomes from *Wolbachia* strains plus 9 outgroup draft genomes. Roary gene clusters without clear gene annotations, listed as “group_#,” were re-examined and if possible assigned gene names by cross-referencing Prokka gene calls to gene and GO annotations for *Wolbachia* strains downloaded from UniProtKB database and other databases (MetaCyc, KEGG). These databases were also used to create a master GO annotation file for Anaplasmataceae for downstream enrichment analyses. Pangenome and core genome comparisons based on Roary gene_presence_absence.csv outputs were depicted with the online Venn drawing tool http://bioinformatics.psb.ugent.be/webtools/Venn/. Functional GO enrichment was assessed using topGO version 2.4.0 (Alexa and Rahnenfuhrer, 2020) which assesses GO-term graph topology ad creates test statistics using the algorithm ‘weight01.fisher’ which returns corrected p-values not affected by multiple testing. TopGO was implemented in R using the script aip_topgo_usage.consider_universe.R^3^ for multiple gene subsets (depicted in Venn diagrams) using ‘diff’ and ‘universe’ gene sets.

### Analysis of Functional Enrichment for Gene Classes With Different dN/dS

To assess genes and gene regions that may be important functionally, through signatures of increased or decreased purifying or directional selection, we applied an analysis approach that assessed pairwise dN/dS followed by GO enrichment tests on various high and low dN/dS gene sets. These analyses involved first generating new pairwise codon-based nucleotide alignments of orthologs generated in Roary, for each pair of *Wolbachia* strains. We then generated sliding window blocks using the function split.java in KaKs Calculator 2.0 (Zhang et al., 2006) to create 1,200 bp length windows with overlaps of 600 bp, preserving codon positions. We then used KaKs Calculator to assess dN/dS on all windows, specifying genetic code 11. This program assesses Ka/Ks (or dN/dS) while controlling for multiple substitutions per site and using a maximum likelihood framework for model selection and AICc for model averaging (Zhang et al., 2006). In addition to multiple substitution corrections performed by this software, we also set a maximum Ks cutoff for our output data of 2 for *Wolbachia* pairs in the major supergroups, and 2.5 for *Wolbachia* in the PPN supergroup. Annotated genes were matched and counted as within windows if they occurred across at least 300 bp of a given window. Output dN/dS values were grouped into subsets for topGO functional enrichment analysis, partitioning genes into sets with values into top 10%, top 25%, the bottom 10%, and bottom 25% of each pairwise comparison.

## Results

### Nematode Communities Were Screened and Found Positive for *Wolbachia*

Screening for PPN-type *Wolbachia* was initially performed on 16 samples (Supplementary Table 1) with the initial screening of raw reads showing 10 out of 16 samples with reads mapping to the *Wolbachia* 16S rRNA gene. However, blastn analysis of these reads showed that most of these read hits were more similar to non-PPN *Wolbachia* strains than to PPN strains, with only one sample (P3-11) showing high similarity to *w*Ppe from the plant-parasitic nematode *P. penetrans* and *w*Rad *Wolbachia* from *R. similis.* This sample was from a fruit tree farm in Los Fresnos, Texas (26.1585 N 97.3844 W) consisting of pooled soils from the following fruit trees: mango (*Mangifera indica*; Sapindales: Anacardiaceae), guava (*Psidium guajava*; Myrtales: Myrtaceae), pomello (*Citrus maxima*; Sapindales: Rutaceae), sugar-apple (*Annona squamosa*; Magnoliales: Annonaceae), and sapodilla (*Manilkara zapota*; Ericales: Sapotaceae). After the discovery of this initial positive result, separate samples were collected from 14 individual fruit trees at the same farm, from which the following five fruit tree samples were found to be PCR-positive and were processed for community shotgun sequencing: sugar-apple, avocado (*Persea americana*; Laurales: Lauraceae), plantain (*Musa* × *paradisiaca* AAB; Zingiberales: Musaceae), guava, and “Rosetta fruit” (*Malus pumila* “Niedzwetzkyana”; Rosales: Rosaceae) (Supplementary Table 1).

### Genome Assembly and General Features of *Wolbachia* Strain *w*Tex

**FIGURE 1:**
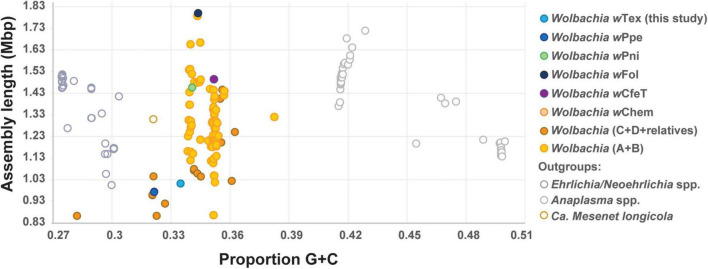
Plot of estimated genome sizes (total assembly lengths) versus proportion G + C content for Anaplasmataceae including *Wolbachia* and outgroups, showing PPN-type strains *w*Tex and *w*Ppe and early-branching clades *w*Pni and *w*Fol, and others.

Based on initial alignments of contigs from six *Wolbachia*-positive samples which originated from the same farm, inter-sample divergence was found to be low, therefore, to increase coverage, final genome assembly was performed for the six samples with reads pooled together. The final assembly (NCBI GenBank accession JAIXMJ000000000) of the new *Wolbachia* strain, hereafter, designated *w*Tex (named for its location in southern Texas), consisted of 192 scaffolds with a total length of 1,013,022 bp, maximum scaffold length of 57,862 bp, N50 of 10,082, with 33.49% GC, coding density 0.809, with 989 predicted genes, and a full set of rRNA and tRNA genes (3 and 38, respectively), and average coverage of 15.96X (Supplementary Table 2). CheckM-based genome completeness was 84.37% based on 368 markers from 63 Rickettsiales genomes, with a contamination score of 0.64 CheckM. Prior to this final assembly, during iterative steps to improve the assembly CheckM completeness scores decreased as contamination scores decreased. Comparative genome features across similar *Wolbachia* strains are shown in Table 1. Among the predicted genes, 398 (40.2%) had no known function. GC content (Figure 1) was intermediate between that of clades C and D *Wolbachia* strains from filarial nematodes and clades A and B from arthropods, whereas assembly length was shorter than that of the majority of clades A and B *Wolbachia* strains and was more similar to that of clades C and D *Wolbachia*.

**TABLE 1:** Comparative gene and genome features for whole-genome shotgun assemblies for the new *Wolbachia* strain, designated *w*Tex, and its closest relatives, *w*Ppe from the plant-parasitic nematode *Pratylenchus penetrans*, *w*Pni from the banana aphid *Pentalonia nigronervosa*, *w*Fol from the springtail *Folsomia candida*, *w*CfeT from the cat flea *Ctenocephalides felis*, *w*Chem from the bedbug *Cimex hemipterus*.

*Wolbachia* strain name	Genome assembly length	%GC	Genes	rRNAs, tRNAs	Pseudo-genes	Contigs	NCBI accession
*w*Tex	1,013,022	33.49	989	3, 38	2	192	JAIXMJ000000000
*w*Ppe	975,127	32.16	962	3, 36	9	12	NZ_MJMG01000001.1
*w*Pni	1,457,187	34.09	1314	3, 37	4	182	JACVWV010000040.1
*w*Fol	1,801,626	34.35	1601	3, 36	11	1	NZ_CP015510.2
*w*CfeT	1,495,538	35.18	1519	3, 35	55	1	NZ_CP051156.1
*w*Chem	1,291,339	35.37	1266	3, 33	41	1	NZ_CP061738.1

*Gene prediction was performed using the same parameters in Prokka to obtain comparable gene predictions among all draft genomes.*

### Correlation Between Nematodes and *Wolbachia*-Positive Samples and Cytochrome Oxidase I Phylogeny of Candidate *w*Tex Hosts

Correlation analyses (Supplementary Figure 1) showed *Wolbachia w*Tex was positively associated with nematode hits to the partial COI gene of Heteroderidae sp. CD2526 (GenBank accession MK033155.1) (rho 0.659044735, BH-corrected *p*-value 0.02339631) and *Ptycholaimellus* sp. M1 (GenBank accession KX951909.1) (rho 0.713184097, BH-corrected *p*-value 0.006335523), however, hits matching the latter were very short and these scaffolds were at very low coverage. Scaffolds identified as matches to the COI of Heteroderidae sp. CD2526 were generally longer > than 1,000 bp with longer blast matches and were therefore extracted for further analysis. This produced 9 scaffolds with similarities to this COI hit. Similar sequences from GenBank were downloaded and aligned and analyzed by ML and Bayesian methods, which showed 7 sequences clustered with support in a clade together with *Helicotylenchus* spp. while 2 sequences clustered in a sister-clade with *Rotylenchus* spp. (Supplementary Figures 2, 3).

### Phylogenetic Analysis of *w*Tex With Other *Wolbachia* Strains and Outgroups

Phylogenetic analyses for the 16S rRNA gene alone (Supplementary Figure 4) which included the broadest array of outgroups and additional early-branching *Wolbachia* in this study and an alignment block length of 1,573 nucleotide positions, resulted in a strongly supported clade for PPN *Wolbachia* strains *w*Tex, *w*Ppe, and *w*Rad, in a basal position in the *Wolbachia* phylogeny. The closest early-branding sister taxa to this PPN *Wolbachia* clade included a well-supported cluster from various aphids including *Pentalonia nigronervosa w*Pni-like strains (accessions NZ_JACVWV01000005.1, KJ786949.1, KJ786950.1) along with strains from the conifer aphid *Cinara cedri* (AY620430.1) and the trunk-feeding aphid *Stomaphis sinisalicis* (KF751211.1), and strains *w*Bta from the whitefly *Bemisia tabaci* (KF454771.1) and *w*Bry from the spider mite *Bryobia* spec. V (EU499316.1). The largest sequence difference in the 16S rRNA region among PPN-type *Wolbachia* strains was 4.211%, whereas the average difference between the PPN-clade and *w*Pni-like strains was 3.934%. A separate early-branching sister clade consisted of closely related isolates of a *Wolbachia* from the fungal-feeding mold mite, *Tyrophagus putrescentiae* (Supplementary Figure 4).

**FIGURE 2:**
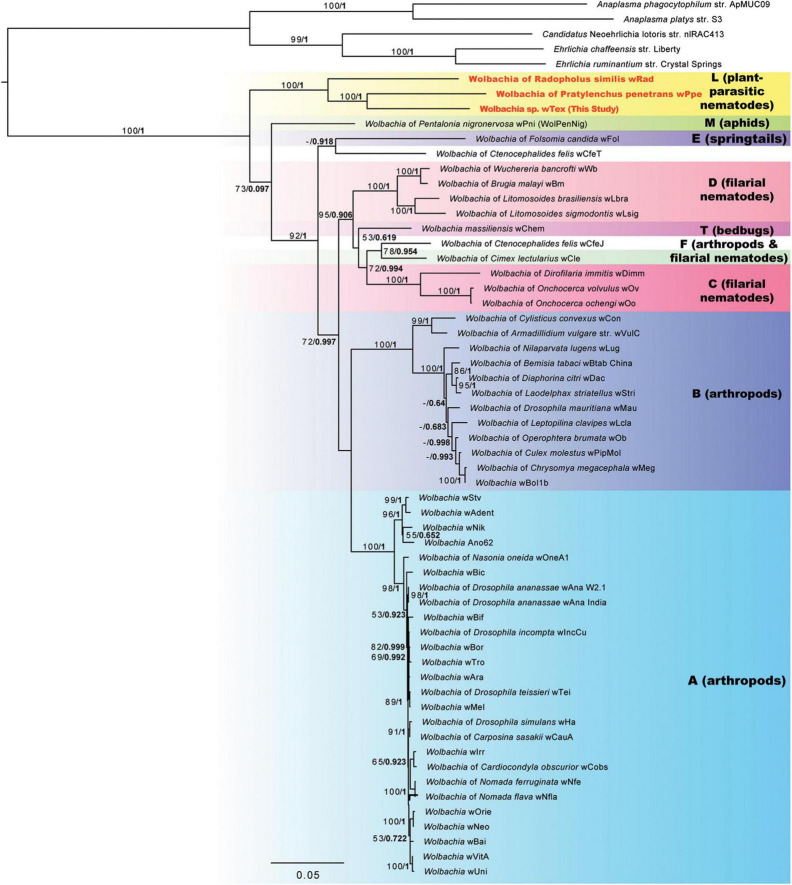
Phylogeny of three gene regions (16S rRNA, CTP synthase/ftsZ, and groES/groEL) for *Wolbachia* and outgroups based on 3,656 aligned positions of the concatenated nucleotides with gaps and ambiguities and the most variable 1/10th of positions removed. Maximum likelihood phylogeny reconstruction was performed in RAxML GTR + Gamma with bootstrap support >50% from 500 replicates shown on branches. Most supported nodes were obtained with high support in Bayesian 50% majority rule in MrBayes with GTR + G with 4 rate categories model. Bayesian posteriors are shown next to bootstrap values, in bold. Sequences obtained in this study are indicated in bold orange font.

Phylogenetic analyses for the concatenation of 3 gene regions (16S rRNA, ftsZ, and groEL) to better resolve the phylogeny including all three PPN-type strains *w*Tex, *w*Ppe, and *w*Rad resulted in a strongly supported clade for PPN *Wolbachia* strains (Figure 2). The supported clade was obtained for both ML and Bayesian analyses, which produced similar tree topologies, and for all alternative alignments including the full sequence alignment, the gap-stripped alignment, and alignments with progressive stripping of high evolutionary rate sites resulting in alignment lengths of 2,368 to 4,674 nucleotide positions. Bootstrap support for the PPN *Wolbachia* clade was 85–100%, with support of 100% for the majority of the alternate alignments. The PPN *Wolbachia* clade formed a strongly supported earliest branch for the genus *Wolbachia* (supergroup L in Figure 2) for alignments with full data, gap-stripped data, and data with high evolutionary rate sites stripped that included over 68% of the original alignment length, however, progressive stripping of such high-rate sites placed strain *w*Pni at the root of the tree with or without >50% bootstrap support and reduced support for other basal branches.

**FIGURE 3:**
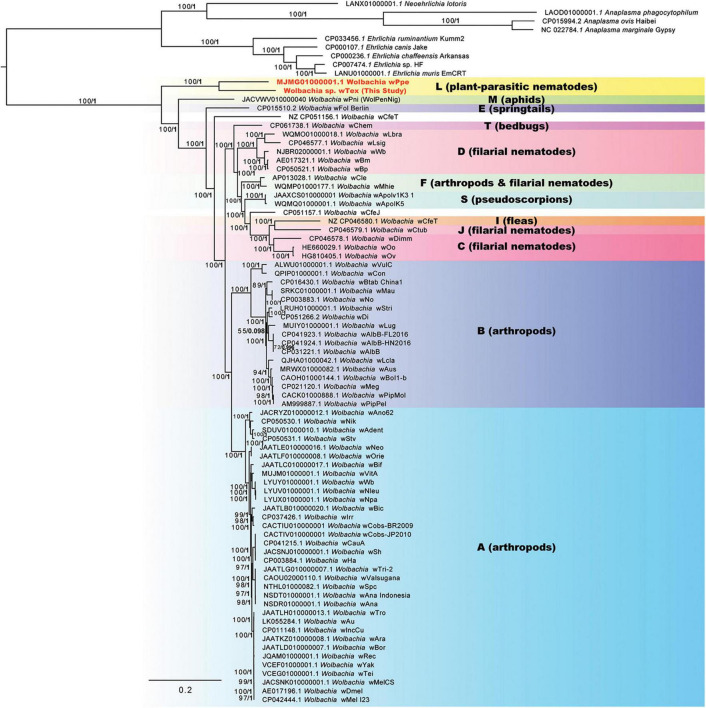
Phylogeny of 100 core protein-coding genes for *Wolbachia* and outgroups based on 29,264 aligned nucleotide positions identified from assemblies using Roary software, with gaps and ambiguous states and 3rd positions of codons removed. Maximum likelihood phylogeny reconstruction was performed in RAxML GTR + Gamma with bootstrap support >50% from 500 replicates shown on branches. Most supported nodes were obtained with high support in Bayesian 50% majority rule in MrBayes with GTR + G with 4 rate categories model. Bayesian posteriors are shown next to bootstrap values, in bold. Sequences obtained in this study are indicated in bold orange font.

Phylogenomic analyses of 100 genome-wide protein-coding genes (orthologs from Roary), resulted in similar results to the 3-region results (above), producing a strongly supported clade for PPN *Wolbachia* strains *w*Tex and *w*Ppe (Figure 3). ML bootstrap support and Bayesian posterior probabilities were largely similar for these analyses, showing support for this clade of 100% bootstrap and posterior of 1. These results were consistent for alternative alignments including the full sequence alignment, the gap-stripped alignment, an alignment with 3^rd^ codon positions removed, a translated alignment, and alignments with progressive stripping of high evolutionary rate sites resulting in alignment lengths of 17,382 to 87,628 nucleotide positions. The exception to this high support was alignments stripped to less than 20% of the original positions, in which there was very little support for any *Wolbachia* clade. As with the 3-region phylogenetic analysis, the 100-gene phylogenetic analyses produced a strongly supported earliest branch position for the PPN *Wolbachia* clade (supergroup L in Figure 3) in some, but not all alternative alignments. For example, for alignments with full data, full data with gaps and ambiguities stripped, and full data with 3rd codon positions stripped, there was 100% bootstrap support for the PPN *Wolbachia* clade forming the earliest branch for *Wolbachia*. However, for alignments with high evolutionary rate sites stripped such that the alignment was 25–43% of its original length, the earliest branch of genus *Wolbachia* varied, sometimes placing *w*Pni at the root of the tree just before PPN *Wolbachia*, and sometimes placing strains *w*Fol and *w*CfeT as sisters to PPN *Wolbachia*, together forming the earliest branch. However, one alignment with high-rate sites stripped, with 22% of the original length, produced 91% bootstrap support for PPN *Wolbachia* at the root of the genus. Phylogenies based on amino acid alignments, however, also placed strain *w*Pni at the root with 82% bootstrap support.

### Screening of *Wolbachia* From Global Soil and Rhizosphere Sequence Read Archive Databases

**FIGURE 4:**
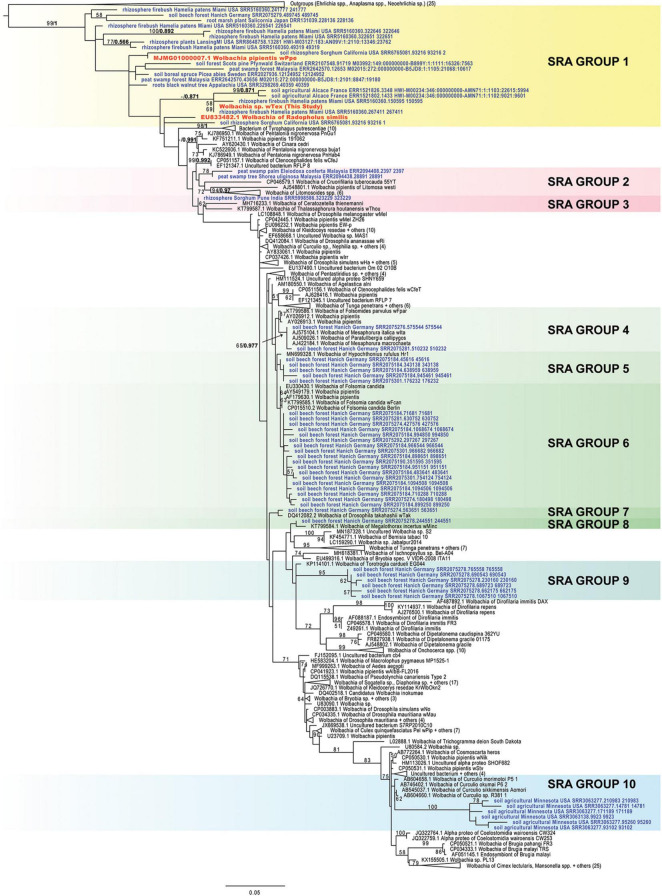
Phylogeny of plant-parasitic nematode-type *Wolbachia*-like matches from the SRA database hits 1,047 bp aligned positions of the 16S rRNA gene. Maximum likelihood phylogeny reconstruction was performed in RAxML GTR + Gamma. Bootstrap support >50% from 500 replicates is shown on branches along with posterior probabilities from Bayesian 50% majority rule in MrBayes with GTR + G with 4 rate categories model, shown for several SRA nodes. Sequences from the SRA are indicated in bold blue font and sequences from PPN *Wolbachia* are indicated in orange bold font. Highlights show clades with *Wolbachia*-like hits.

Sequence read archive (SRA) database screening of 3,400 amplicon experiments from soils and rhizospheres produced 81 sequence runs (i.e., SRR/ERR/DRR files) with the highest blastn similarity to the 16S rRNA gene from PPN-type *Wolbachia w*Tex, *w*Ppe, or *w*Rad. Following read-merging, there was 4,535 top sequences read blastn matches to PPN-type *Wolbachia* among these runs. After removal of identical sequence reads and sequences leading to exceptionally long branches in phylogenies and sequences placed ambiguously between the *Ehrlichia*/*Anaplasma* and *Wolbachia* clades, there were 61 unique sequences unambiguously grouped with the *Wolbachia* clade. These sequences were from 24 separate SRA runs originating from the United States, France, Germany, Sweden, Switzerland, Japan, India, and Malaysia. Although phylogenetic analyses with these short sequences produced generally low bootstrap support and low posterior probabilities, tree topologies suggest several clades (Figure 4). Group 1 comprised 19 sequences clustered at the root of the *Wolbachia* tree along with *Wolbachia* from PPNs with broad geographic origins (France, Germany, Sweden, Switzerland, Japan, Malaysia, and in the United States, from Florida, Michigan, California, Appalachia) from diverse ecosystems. Groups 2 and 3, from Malaysia and India, clustered near the root of the *Wolbachia* CDF supergroups. Groups 4 through 8, comprising 28 sequences, were all from a beech forest in Germany and sequences clustered mostly with various *Wolbachia* strains from Collembola. Group 9 was a distinct cluster of 6 sequences from the same beech forest with similarity to *Wolbachia* from quill mites, while Group 10 formed a cluster of 6 sequences allied with *Wolbachia* from *Curculio* sp. (weevils) from Minnesota. Phylogenetic trees for separate sub-regions produced similar results (Supplementary Figures 5–7).

### Predicted Genes and Pathways Present, Absent, or Unique to Plant-Parasitic Nematode-Type *Wolbachia*

Specific predicted pathways and genes of interest based on known or hypothesized functions were searched for in the *w*Tex assembly. The *w*Tex genome was similar to *w*Ppe and other *Wolbachia* in having conserved pathways for glycolysis and the tricarboxylic acid cycle and pathways for biosynthesis of nucleotides including the pentose phosphate pathway, and peptidoglycan and fatty acids, but lacking genes for key steps or most steps of other biosynthetic processes including amino acid, vitamin and co-factor, and carbohydrate synthesis, suggesting incomplete pathways. Both PPN-type *Wolbachia* strains *w*Tex and *w*Ppe had full-length predicted genes for heme synthesis that were also conserved across outgroups and other *Wolbachia*, including *hemA* (encoding 5-aminolevulinate synthase EC 2.3.1.37), *hemB* (encoding delta-aminolevulinic acid dehydratase EC 4.2.1.24), *hemC* (encoding porphobilinogen deaminase EC 2.5.1.61), *hemE* (encoding uroporphyrinogen decarboxylase EC 4.1.1.37), *ctaB* (encoding protoheme IX farnesyltransferase EC 2.5.1.141), *hemF* (encoding oxygen-dependent coproporphyrinogen-III oxidase), and *hemH* (encoding ferrochelatase EC 1.3.3.3).

Riboflavin synthesis and transport genes were notably absent in *w*Tex and *w*Ppe, except for *ribB* (encoding 3,4-dihydroxy-2-butanone 4-phosphate synthase EC 4.1.99.12) and one outstanding gene annotated as *ribN* (riboflavin transporter) in *w*Ppe that was not present in any *Wolbachia* or outgroup strains. Similarly, biotin synthesis genes were absent in *w*Tex and *w*Ppe, with no homologs found matching either outgroup Anaplasmataceae-type biotin genes or the frequently horizontally transferred ‘BOOM’ (biotin synthesis operon of obligate intracellular microbes) operon genes, *bioA* (encoding adenosylmethionine-8-amino-7-oxononanoate aminotransferase EC 2.6.1.62), *bioB* (encoding biotin synthase EC 2.8.1.6), *bioC* (encoding malonyl-[acyl-carrier protein] *O*-methyltransferase EC 2.1.1.197), *bioD* (encoding ATP-dependent dethiobiotin synthetase EC 2.6.1.62), *bioF* (encoding 8-amino-7-oxononanoate synthase 2 EC 2.3.1.47), and *bioH* (encoding pimeloyl-[acyl-carrier protein] methyl ester esterase EC 3.1.1.85). However, both *w*Tex and *w*Ppe assemblies had a predicted *bioY* gene (encoding a biotin importing transporter protein), and *birA* (encoding the biotin ligation protein bifunctional ligase/repressor EC 6.3.4.15), and diverged variants of the biotin utilization genes *pccB* (encoding propionyl-CoA carboxylase beta chain EC 2.1.3.-) and *fabD* (encoding malonyl CoA-acyl carrier protein transacylase EC 2.3.1.39). The latter two genes had such low sequence similarity to other *Wolbachia* strains and outgroups that they were not clustered as homologs to similarly annotated copies in Roary.

**FIGURE 5:**
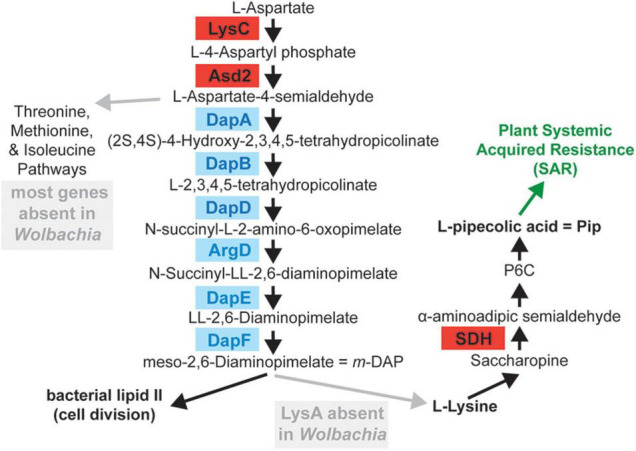
Lysine biosynthesis pathway (diaminopimelate or DAP pathway) and lysine catabolism to pipecolic acid (Pip) showing predicted enzymes that are conserved among plant-parasitic nematode (PPN) *Wolbachia*, with predicted horizontally transferred genes (HGTs) in red. The genes for LysC and Asd2 genes reflect a putative *Midichloria*-like HGT, whereas the gene for SDH reflects a putative eukaryote-like HGT to PPN-*Wolbachia* and also the related early-branching strain *w*Pni. Gray boxes show missing steps likely filled by plant-to-nematode-to-*Wolbachia* supplementation and gray arrows point to intermediates that could be secreted by PPN-*Wolbachia* to the host.

Additional predicted genes in *w*Tex that were shared between PPN-type *Wolbachia w*Tex and *w*Ppe, but unique to these strains (i.e., not found in other *Wolbachia*) included a 1,350 nt gene *lysC* (encoding lysine-sensitive aspartokinase 3 EC 2.7.2.4, involved in the first step of the lysine biosynthesis *via* diaminopimelate ‘DAP’ pathway (see Figure 5) which also leads to methionine biosynthesis *via de novo* pathway, and threonine biosynthesis). This gene had the closest blastn and blastx similarity to genes from the alphaproteobacteria *Candidatus* Midichloria mitochondrii (hereafter *Midichloria*) (accession NC_015722.1), or distant genera of Vibrionales (*Photobacterium*, *Vibrio*), but no homologs to the standard *Wolbachia* or Anaplasmataceae variants of the *lysC* gene. Directly adjacent to this predicted *lysC* gene was a unique 987 nt PPN-type *Wolbachia* variant of *asd2* (encoding aspartate-semialdehyde dehydrogenase 2 EC 1.2.1.11, which catalyzes the second step in lysine biosynthesis *via* the DAP pathway), with closest blast matches to genes from *Midichloria* and next to distantly related bacteria *Photobacterium*, and *Vibrio*. Although *w*Ppe had both variants of *asd2*, *w*Tex had only the *Midichloria*-like variant. These *Midichloria*-like *asd2* and *lysC* genes were located adjacent to *Wolbachia*-like *carA* (encoding carbamoyl-phosphate synthase small chain EC 6.3.5.5, the first step of pyrimidine and arginine synthesis) and *dapA* (encoding 4-hydroxy-tetrahydrodipicolinate synthase EC 4.3.3.7, catalyzing the third step in the lysine biosynthesis pathway). The *asd2* and *lysC* gene operon was syntenic, with the same gene order and orientation as that of *Midichloria*, although the latter had a branched-chain amino acid transaminase (BCAT EC 2.6.1.42) in the place of the *dapA* in PPN-type *Wolbachia*, whereas the distantly related versions of *asd2* and *lysC* in other *Wolbachia* strains and outgroup Anaplasmataceae were not located together in tandem (Supplementary Figure 8). Phylogenetic analyses of all *asd2* and *lysC* variants showed that the PPN-type *Wolbachia* and *Midichloria* versions of these genes have phylogenetically diverged from all other Anaplasmataceae/*Wolbachia* variants with high bootstrap support for a sister-clade to *Midichloria* for both genes (Supplementary Figures 9, 10). Nucleotide identity between *Midichloria*-like and PPN-type *asd2* and *lysC* genes was ∼63 and 60%, respectively, while identity between *w*Tex and *w*Ppe homologs were ∼79 and 81%, respectively. Given the finding of these lysine biosynthesis pathway genes (Figure 5), and the observation that the missing final gene required for lysine synthesis *lysA* (encoding diaminopimelate decarboxylase EC 4.1.1.20) was previously predicted as a bacteria-to-eukaryote HGT in whiteflies (Luan et al., 2015) we searched the complete metagenome assemblies comprising mostly nematode contigs using blastn and blastp to see if we could detect *lysA* in a contig matching nematodes, but no such match was found. However, coverage was low, leaving some uncertainty about missing genes.

Another predicted lysine metabolism gene, shared only among PPN-type *Wolbachia* (wTex_00187) and *w*Pni, was most similar (67% amino acid identity) to a saccharopine dehydrogenase (SDH) gene (EC 1.5.1.9) in the conifer aphid (*Cinara cedri*), involved in lysine catabolism (Figure 5). There was also synteny conservation in *w*Tex and *w*Ppe for a 1,308 nt predicted gene (wTex_00248) which had no blastn or blastx matches in the nt/nr databases, which was adjacent to *hemA*, which catalyzes the first step of heme synthesis. Another unique gene in *w*Tex and *w*Ppe was a 1,494 nt predicted gene *tlcA* (encoding ADP, ATP carrier protein 1) with its closest match being 70% nucleotide similarity to homologs in *Rickettsia*. There was a 729 nt predicted gene with partial blastn similarity to a squalene/phytoene synthase/isoprenoid synthase gene (potentially involved in carotenoid synthesis) from the conifer aphid *C. cedri* and a *Wolbachia* strain from the gall mite *Fragariocoptes setiger*. The latter was adjacent to *dxr* (encoding 1-deoxy-D-xylulose 5-phosphate reductoisomerase EC 1.1.1.267). Two other shared genes predicted in *w*Tex and *w*Ppe were arrayed in synteny: a 663 nt gene annotated as *deoC* (a gene involved in carbohydrate degradation EC 4.1.2.4) with high blastx similarity to BON (bacterial OsmY and nodulation) domain-containing protein genes in unrelated bacteria such as *Flavobacteria*, *Holosporaceae*, *Cardinium*, and *Rickettsia*; and an adjacent 246 nt predicted gene with DUF2188 domain and no matches in either blastn or blastx to the nt/nr databases. The first of these two genes had partial length homology (69–80% amino acid identity) to genes from two *Wolbachia* strains, *w*CfeT from cat fleas and a strain from the gall mite *F. setiger*. There were also predicted *def* (encoding peptide deformylase EC 3.5.1.88) and pterin-4-alpha-carbinolamine dehydratase genes (PCBD1 EC 4.2.1.96) involved in phenylalanine metabolism to tyrosine through tetrahydrobiopterin with distinct homologs in PPN-type *Wolbachia* that more closely matched non-*Wolbachia*, including eukaryotes (*Culicoides* midges) and distantly related bacteria (*Francisella*), or *Rickettsia* and an ameba endosymbiont (*Candidatus* Nucleicultrix amoebiphilia), respectively.

We searched for cytoplasmic incompatibility factor genes, *cifA* and *cifB.* From the 92 *Wolbachia* genomes analyzed in Roary, there were 152 *cifA* and *cifB*-like genes identified, yet these shared no homology with any predicted genes in *w*Tex or *w*Ppe or clade C or D *Wolbachia*. Next, we searched for WO prophage-like or plasmid-associated genes. Strain *w*Tex showed no homology to the more than 2,000 phage or prophage-type genes detected in these 92 *Wolbachia* genomes analyzed. Nor did *w*Tex have any detected homologs to the 84 plasmid-type genes or in this set of genomes. Other enriched features of PPN-type *Wolbachia* are described in the section below.

### Comparative Genome Repertoires and Gene Ontology Enrichment Analysis

Analysis of gene repertoire overlap between PPN-type *Wolbachia* strains *w*Tex and *w*Ppe showed 501 predicted shared genes between these strains with an additional 400 and 437 genes only found in *w*Tex and *w*Ppe, respectively. Compared to *w*Ppe, strain *w*Tex was enriched for GO processes DNA-transposition, thiamine biosynthesis, and thiamine diphosphate biosynthesis (Supplementary Table 3), whereas the *w*Ppe strain was enriched for GO processes mismatch repair and one-carbon metabolism (Supplementary Table 4). The thiamine enrichment in *w*Tex arises from three genes, *thiE* (encoding thiamine-phosphate synthase EC 2.5.1.3), *thiM* (encoding hydroxyethylthiazole kinase EC 2.7.1.50), and *thiD* (encoding hydroxymethylpyrimidine/phosphomethylpyrimidine kinase EC 2.7.1.49 EC 2.7.4.7) which occurred as duplication in two scaffolds. Of these genes, only *thiM* was present in *w*Ppe, whereas the variants of these genes in *w*Tex had close matches only to one *Wolbachia* strain (*w*CfeT) and otherwise were most similar to these genes in the spirochete *Brachyspira*.

**FIGURE 6:**
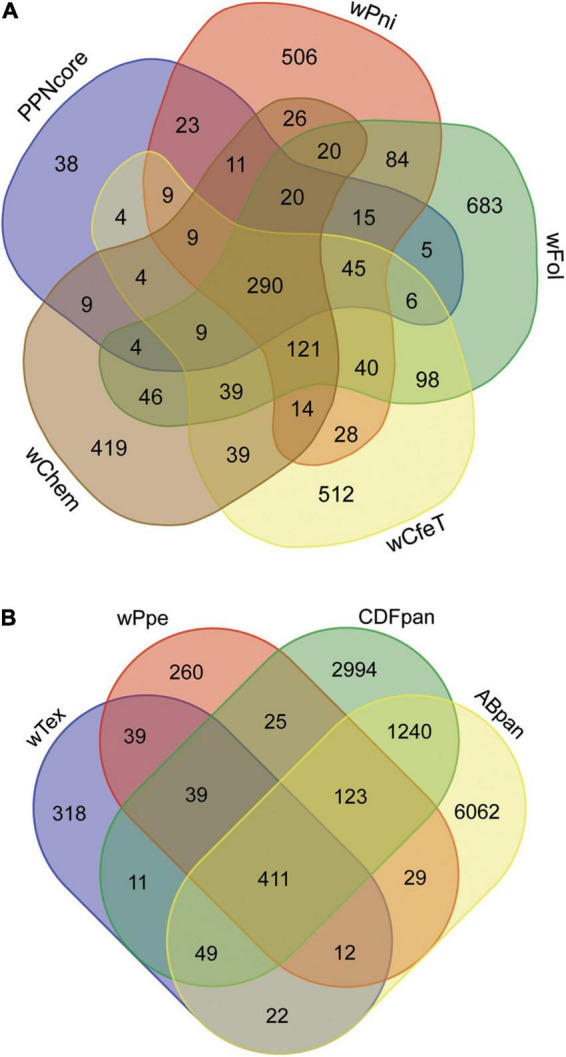
Gene content shared among *Wolbachia* strains from plant-parasitic nematodes (PPNs) *w*Tex and *w*Ppe, compared with other *Wolbachia* clades. **(A)** Depicts core shared genes from PPN *Wolbachia* compared with pangenomes from other early-branching strains, *w*Pni from the banana aphid *Pentalonia nigronervosa*, *w*Fol from the springtail *Folsomia candida*, *w*CfeT from the cat flea *Ctenocephalides felis*, *w*Chem from the bedbug *Cimex hemipterus*. **(B)** Depicts shared genes among PPN strains *w*Tex and *w*Ppe compared with members of the most widespread supergroups A and B (ABpan), and supergroups with representatives from nematodes, supergroups C, D, and F (CDFpan).

Comparison of the shared predicted genes between these two strains (i.e., core genes) compared to the four most closely related strains with complete genome assemblies (namely, *w*Pni from the banana aphid *Pentalonia nigronervosa*, *w*Fol from the springtail *Folsomia candida*, *w*CfeT from the cat flea *Ctenocephalides felis*, *w*Chem from the bedbug *Cimex hemipterus*), not counting the 290 universally shared genes among these strains, showed the core PPN-type *Wolbachia* gene repertoire was most similar to that of *w*Pni, followed by *w*Fol, then *w*CfeT, and *w*Chem (Figure 6A). This mirrored their phylogenetic places described above, with the four strains having 132, 104, 86, and 66 shared genes, respectively, not counting the 290 universally shared genes between all these strains. GO enrichment for the set of shared core PPN-type *Wolbachia* genes not shared with these other strains or shared with at most two other strains (Figure 6A) showed enrichment for biological processes diaminopimelate, pseudouridine, and chorismite biosynthesis, gluconeogenesis, and lysine biosynthesis *via* diaminopimelate, while GO enriched metabolic processes were metallo-aminopeptidase activity, lyase activity, monooxygenase activity, and ATP binding (Supplementary Table 5).

Comparison of genome repertoires of PPN-type *Wolbachia* strains *w*Tex and *w*Ppe to that of the pangenomes of remaining major supergroup clusters (supergroups C, D, and F denoted “CDFpan” and supergroups A and B denoted “ABpan”) (Figure 6B) showed more core PPN *Wolbachia* genes shared with CDFpan than with ABpan (683 vs. 649, respectively). Specifically considering genes shared with only CDFpan or ABpan, there was a similar pattern (i.e., 39 for CDFpan vs. 12 for ABpan). The AB pangenome was larger than the CDF pangenome (7,848 vs. 4,892 genes, respectively), therefore alternatively, the differences could be calculated as proportions of each pangenome cluster shared with PPN-type *Wolbachia*. The latter comparison showed PPN-type *Wolbachia* shared 0.1396 of their pangenome with CDFpan whereas 0.0817 of their pangenome with ABpan. Conversely, there were more accessory genes (not shared between PPN-type *Wolbachia* strains) that were shared with ABpan than with CDFpan (22 vs. 11 for *w*Tex and 29 vs. 25 for *w*Ppe), although this difference was not found when controlling for the approximately doubled number of accessory (non-shared) genes in ABpan compared with CDFpan (6,062 vs. 2,994 genes, respectively).

**FIGURE 7:**
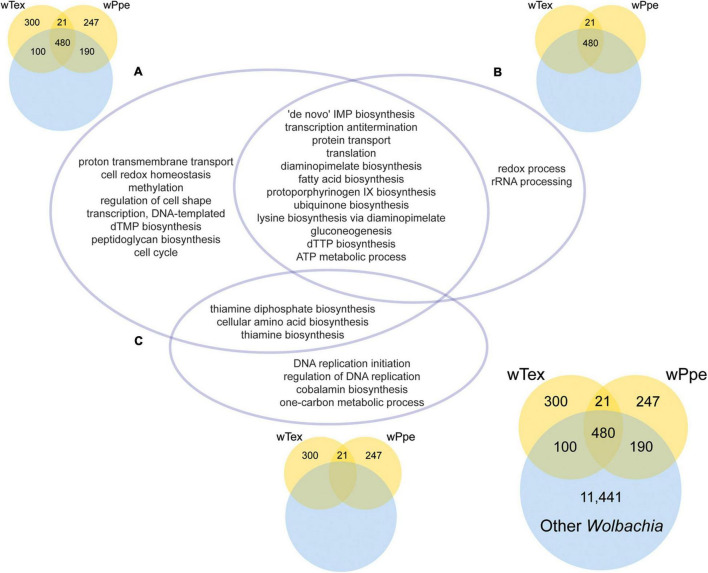
Significantly enriched gene ontology (GO) categories for the pangenomes of plant-parasitic nematode-associated *Wolbachia* strains (*w*Tex and *w*Ppe) compared to other *Wolbachia*. The intersections of three GO enrichment tests **(A–C)** are depicted in the central Venn diagram. **(A)** Corresponds to the colored Venn on the top left depicting GO enrichment for the PPN pangenome including genes shared with other *Wolbachia*. **(B)** Corresponds to the colored Venn on the top right depicting GO enrichment for core shared PPN genes including those shared with other *Wolbachia*. **(C)** Corresponds to the colored Venn on the bottom middle depicting GO enrichment for PPN pangenome genes not shared with other *Wolbachia*. The bottom right Venn shows numbers of genes compared for all groups. Full topGO results are shown in Supplementary Tables 6–8.

The GO enrichment analyses for PPN-type *Wolbachia* strains *w*Tex and *w*Ppe compared to other *Wolbachia* showed several significantly enriched functions (Figure 7 and Supplementary Tables 6–8). GO enrichment was compared for three sets of overlap (Figure 7), first for PPN-type *Wolbachia* compared to the pangenome of all *Wolbachia*, including genes shared between pangenomes (Figure 7A and Supplementary Table 6), then for the core shared genes from PPN-type *Wolbachia* compared to the pangenome of all *Wolbachia* (Figure 7B and Supplementary Table 7), then for the total pangenomes of PPN-type *Wolbachia* compared to the pangenome of all *Wolbachia* (Figure 7C and Supplementary Table 8). Among PPN-type *Wolbachia*, there was significant GO term enrichment (Figure 7A) for various cellular biosynthetic processes, including several nutrient pathways: protoporphyrinogen IX biosynthesis (part of heme synthesis), thiamine and thiamine diphosphate biosynthesis, lysine biosynthesis *via* diaminopimelate and diaminopimelate biosynthesis, fatty acid biosynthesis, cellular amino acid biosynthesis. Among GO enrichment for core functions shared by *w*Tex and *w*Ppe (Figure 7B) were many GO terms including nutrient pathways such as protoporphyrinogen IX biosynthesis, lysine biosynthesis *via* diaminopimelate and diaminopimelate biosynthesis, and fatty acid biosynthesis. For pangenomes of *w*Tex and *w*Ppe excluding shared genes with other *Wolbachia* (Figure 7C), enriched GO terms included thiamine and thiamine diphosphate biosynthesis, cellular amino acid biosynthesis, and cobalamin biosynthesis.

### Analysis of Changes in Signatures of Selection on Gene Ontology Categories Across Early-Branching *Wolbachia* Strains

Analysis of dN/dS to investigate signatures of purifying or positive selection within PPN-type *Wolbachia* (*w*Tex and *w*Ppe) produced a range of dN/dS values from 0.0119336 to 0.334742, with a mean dN/dS of 0.08011301, considering values with *K*s < 2. For genes within the dN/dS alignment block that fell within the top or bottom 10% or 25%, topGO enrichment analyses were performed against the pangenome of PPN-type *Wolbachia* (Supplementary Figure 11 and Supplementary Tables 9–12). Enriched GO categories for the highest values of dN/dS indicative of lower-than-average purifying selection included transcription antitermination, protein folding and transport, and various nutrient metabolism processes including arginine metabolism, N2-acetyl-L-ornithine:2-oxoglutarate 5-aminotransferase activity (a part of arginine metabolism), diaminopimelate biosynthesis (a lysine precursor), lysine biosynthesis, pyridoxal phosphate (vitamin B6) binding (Supplementary Tables 9, 11). Notable GO categories with the lowest dN/dS values (indicative of highest purifying selection) included translation and key metabolic functions associated with an energy (e.g., ATP binding and TCA) and metal ion, iron-sulfur cluster, and heme-binding (Supplementary Tables 10, 12).

**FIGURE 8:**
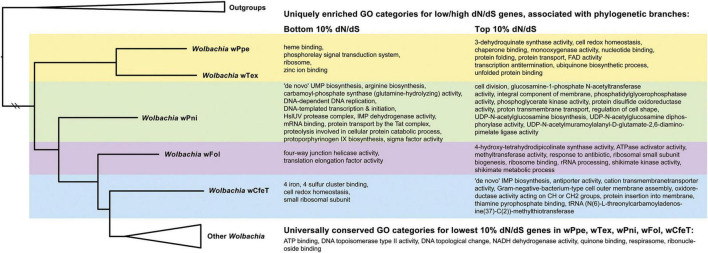
Enriched gene ontology (GO) categories associated with transitions between early-branching clades of *Wolbachia* showing GO enrichment for highest and lowest 10% of dN/dS values for genes from pairs of strains *w*Tex, *w*Ppe, *w*Pni, *w*Fol, and *w*CfeT. Pairwise dN/dS was calculated with KaKs Calculator for overlapping 1,200 bp blocks and GO enrichment was calculated with topGO at *p*-value < 0.05 and reported for unique GO terms among clades as well as universally conserved (bottom 10%) values. Shading: yellow = unique to plant-parasitic nematode-associated *Wolbachia*, green = unique to plant-parasitic nematode-associated *Wolbachia* and *w*Pni, purple = unique to *w*Pni and *w*Fol, blue = unique to *w*Fol and *w*CfeT. Full topGO results are shown in Supplementary Tables 13–20.

The analysis of enriched GO categories for gene sets with highest and lowest dN/dS values associated with transitions between early-branching clades of *Wolbachia* was analyzed (Figure 8 and Supplementary Tables 13–20). Specifically, universally conserved lowest dN/dS (bottom 10% values) shared among early-branching clades of *Wolbachia* (strains *w*Tex, *w*Ppe, *w*Pni, *w*Fol, and *w*CfeT – branch topology and branch lengths extracted in Figure 8 from Figure 3) showed 7 GO categories including DNA topoisomerase type II activity and respirasome. In contrast, conserved lowest dN/dS gene sets showed diverse differences in GO terms unique to each branch. PPN-type *Wolbachia* strains *w*Tex and *w*Ppe showed unique (not shared) enrichment for heme binding, phosphorelay signal transduction system, ribosome, and zinc ion binding. Uniquely enriched GO terms from the lowest dN/dS gene set in the branch from PPN-type *Wolbachia* to *w*Pni included 12 terms including protoporphyrinogen IX biosynthetic process. Uniquely low dN/dS gene set GO enrichment in the branch from *w*Pni to *w*Fol included four-way junction helicase and translation elongation factor activity, while the respective enriched terms from *w*Fol to *w*CfeT were 4 iron, 4 sulfur cluster binding (Figure 8). Various GO categories were uniquely enriched for the highest dN/dS gene sets (top 10%) in these branches, such as for PPN-type *Wolbachia*, in terms of protein folding and transport, chaperone binding, and 3-dehydroquinate synthase activity (part of aromatic amino acid synthesis *via* the shikimate pathway) (Figure 8). For the branch from PPN-type *Wolbachia* to *w*Pni, high dN/dS enrichment included glucosamine-1-phosphate *N*-acetyltransferase activity (involved in amino sugar metabolism) and many cellular membranes and cell shape/division terms. For the branch from *w*Pni to *w*Fol, high dN/dS enrichment included 4-hydroxy-tetrahydrodipicolinate synthase activity (involved in lysine biosynthesis) and various ribosome and rRNA processing functions, as well as shikimate metabolism (Figure 8). For the branch from *w*Fol to *w*CfeT, high dN/dS enrichment included 7 terms (Figure 8), including thiamine pyrophosphate binding (involved in thiamine transport).

## Discussion

While *Wolbachia* is widespread in insects and filarial nematodes, its presence and importance in rhizosphere hosts are largely unknown. Its distribution and function in plant-parasitic nematodes (PPNs) are of interest evolutionarily, ecologically, and for agriculture as a potential target for developing new biological controls. Here, we screened rhizosphere communities for *Wolbachia* strains to gain insight into their presence globally. We discovered, assembled, and analyzed a new PPN *Wolbachia* strain (*w*Tex), comparing functional enrichment and signatures of selection and evaluated genes and genomic patterns that might indicate their role and illuminate their place in the early evolution of this widespread endosymbiont.

Genome features and phylogenetic position of strain *w*Tex confirmed the deep branching place of PPN-type *Wolbachia* (i.e., supergroup L). Profiling of *w*Tex showed it to be similar to *w*Ppe from the PPN *P. penetrans* with respect to important features, such as lacking gene matches to cytoplasmic incompatibility (CI) systems (*cifA* and *cifB*), WO prophages, plasmid-associated genes, and riboflavin (vitamin B2) genes except for *ribB*, the horizontally transferred biotin (vitamin B7) ‘BOOM’ operon, yet possessing biotin import and utilization genes (*bioY*, *birA*, *pccB*, and *fabD*). These features support the hypotheses proposed previously that these widespread, critically important *Wolbachia* host-impacting traits arose later, after the emergence of PPN *Wolbachia via* horizontal gene transfers (HGTs) (Nikoh et al., 2014; Moriyama et al., 2015; Brown et al., 2016; Gerth and Bleidorn, 2016; Chen et al., 2020). We found that although supergroup L *Wolbachia* shared more features (e.g., GC content, assembly length, gene repertoire) with supergroups C, D, and F (comprising mostly obligate mutualists) compared to supergroups A and B, their apparent low prevalence, low titer, suggested by the current study, and possible sex ratio bias phenotype indicated in a previous study (Wasala et al., 2019), suggest they are neither obligate mutualists nor typical CI-inducers. However, our data indicated more unshared than shared genes between PPN *Wolbachia* strains with a large portion of predicted proteins having unknown functions. Despite this open mystery on these accessory genes, our analyses of the shared genes and annotated portions of these genomes yielded insights into the possible function of these *Wolbachia*.

Strains *w*Tex and *w*Ppe showed a core gene repertoire most like that of *Wolbachia w*Pni from the plant-feeding host, banana aphid *Pentalonia nigronervosa*. Furthermore, the closest relatives to PPN-type *Wolbachia* were from 5 plant-feeding specialist arthropod hosts (genera *Pentalonia*, *Stomaphis*, *Cinara*, *Bemisia*, and *Bryobia*), based on available 16S rRNA sequences. Together, these data are consistent with the hypothesis that the earliest *Wolbachia* symbiosis emerged in plant-feeders, raising the question of whether plant diet specialization drove the early emergence of *Wolbachia* prior to the later acquisition of genes, such as nutrient supplementation and cytoplasmic incompatibility genes that led to the widespread success of this symbiosis in non-plant feeding hosts. An obstacle to evaluating this hypothesis is that the role of the *Wolbachia* strains in these plant-specialist hosts remains unclear: previous analysis of *w*Pni suggested it cooperated in nutritional supplementation as a co-mutualist with the aphid primary symbiont *Buchnera* (De Clerck et al., 2014, 2015), but more recent re-analysis questions this idea (Manzano-Marín, 2020). Nevertheless, despite apparently incomplete essential nutrient biosynthesis pathways in PPN *Wolbachia*, the possibility remains that intermediate or missing genes that complete these pathways could be present in the nematode hosts, for example, derived from ancient horizontal gene transfers as observed in the *Portiera*-whitefly symbiosis (Luan et al., 2015; Ankrah and Douglas, 2018). Such HGTs to hosts could also derive from ancient or ancestral bacterial infections (McNulty et al., 2010; Dunning Hotopp, 2011; Brelsfoard et al., 2014; Koutsovoulos et al., 2014; Husnik and McCutcheon, 2018). However, a detailed study of this possibility will require quality PPN genomes and transcriptomes. Nevertheless, the idea that plant diet specialization drove the *Wolbachia* symbiosis fits with estimated fossil-calibrated *Wolbachia* divergence (Gerth and Bleidorn, 2016). Approximating ∼216 million years per 2.8% 16S rRNA gene divergence strains *w*Tex, *w*Ppe, and *w*Rad may have diverged from other *Wolbachia* approximately 314–324 Mya during the high-oxygen mid-Carboniferous period at a time of major nematode diversification, while the *w*Pni-clade *Wolbachia* may have diverged during the major radiation of insects ∼303 Mya (Rota-Stabelli et al., 2013; Brown et al., 2018).

Connected with this pattern of plant-feeding, our GO enrichment analyses hinted at changes in the plant-limited amino acid lysine in the earliest-branching *Wolbachia*. Lysine is an essential amino acid that nematodes cannot synthesize *de novo* but must obtain from their diets, whereas lysine is usually one of the most limiting amino acids in plant diets (Galili, 2002; Galili et al., 2016). Plant roots on which PPNs feed may have especially limited lysine levels due to either lysine catabolism for energy during carbon starvation (Galili et al., 2016), or limited lysine transport to root tissues from the major source in chloroplasts of leaves, or defensive downregulation of lysine synthesis or upregulation of lysine catabolism in roots in response to nematode infection (Pratelli and Pilot, 2014). Thus, one nutrient that PPN-type *Wolbachia* might be expected to supplement is lysine. Consistent with this idea, we found core genes of PPN-type *Wolbachia* were significantly enriched for lysine biosynthesis, while the phylogenetic branch from *w*Pni to *w*Fol (representing a transition from *Wolbachia* in plant-specialists to non-plant specialists) showed decreased purifying selection on lysine biosynthesis (through 4-hydroxy-tetrahydrodipicolinate synthase activity). However, *Wolbachia* appears to lack the final enzyme required for lysine synthesis, *lysA* (encoding meso-diaminopimelate decarboxylase), and our analyses found no homolog of *lysA* in *w*Tex, *w*Ppe, nor any evidence of a *lysA*-like HGT to their respective hosts. Rather than synthesizing lysine, *Wolbachia* is thought to use amino acids, including lysine, as a primary energy source (Wu et al., 2004; Foster et al., 2005; Caragata et al., 2016; Ju et al., 2017; Jiménez et al., 2019), thus, the enrichment for lysine pathway genes could reflect increased demand for intermediates (Figure 5), or could implicate an as yet unknown pathway or pathway complementation route to supplement lysine.

Surprisingly, we found PPN-type *Wolbachia* possessed a conserved *Midichloria*-like putative HGT for lysine biosynthesis genes. We suggest three alternative hypotheses for this unique dap operon (genes *asd2-lysC-dapA*) with putative HGTs *asd2* and *lysC* (hereafter, *asd2-lysC*-HGT). First, the *asd2-lysC*-HGT could compensate for a prior loss of Anaplasmataceae-like versions *lysC* found gene to be missing in both PPN-type *Wolbachia* strains. Curiously, an Anaplasmataceae-like version of *asd2* remained in *w*Ppe but was absent in *w*Tex, leaving open the question of the order of loss and gain of the *asd2-lysC*-HGT and corresponding outgroup genes. Sequencing further strains of early-branching *Wolbachia* could help address this question. A second possibility is that the genes may serve in some unique manner in PPN-type *Wolbachia* to augment or affect the late lysine pathway product, meso-diaminopimelate (*m*-DAP), which is a major constituent of lipid II, which is essential for *Wolbachia* cell division (Vollmer et al., 2013) (Figure 5). A third possibility is that this conserved *asd2-lysC*-HGT generates intermediates that are secreted and transferred amongst compartments of the intimate tripartite association of *Wolbachia* within nematodes within plant roots. Notably, hosts of *w*Ppe and *w*Rad are migratory endoparasites, as are some *Helicotylenchus*, the possible host of *w*Tex. In this scenario, the secreted intermediates could act as substrates in any aspartate-derived amino acid synthesis, including lysine, in the *Wolbachia*-PPN-plant tissue niche. Supporting this secretion idea are data suggesting effector and protein secretion from *Wolbachia* to host cells (Lindsey, 2020) and joint regulation of such secretion systems by host and *Wolbachia* (Li and Carlow, 2012), as well as evidence that PPN *Wolbachia* may be localized with the nematode esophageal glands (Brown et al., 2016, 2018), which are specially modified to secrete hundreds of effectors into plant tissues (Vieira and Gleason, 2019). Interestingly, these early pathway enzymes LysC and Asd2 in the aspartate-derived amino acid biosynthesis pathway are highly inducible (Rodionov et al., 2003; Nærdal et al., 2011) and mutational variants in these genes can significantly increase pathway products (Xu et al., 2016). Such controlled intermediate “nutrient factories” from endosymbionts using horizontally transferred genes from other bacteria, including in some cases, missing dap operon genes, *lysA*, or BCAT (branched-chain amino acid transaminase) have been reported (Luan et al., 2015; Ankrah and Douglas, 2018). Curiously, we found that the *asd2-lysC*-HGT was adjacent to BCAT in *Midichloria* but was adjacent to the lysine pathway gene *dapA* in PPN-type *Wolbachia*, matching the gene order of distantly related *Bacillus* spp. and chlamydiae (Rodionov et al., 2003; McCoy et al., 2006). Unexpectedly, this *dapA* variant was highly similar to the *dapA* gene in *Wolbachia* and other Anaplasmataceae, with its gene tree mirroring the species tree, suggesting that this dap operon gene arose earlier in these alphaproteobacteria.

However, the presence of a second lysine-related putative HGT, the eukaryote-like gene *sdh* for saccharopine dehydrogenase (SDH), in both PPN-type *Wolbachia* and the early-branching relative from aphids, *w*Pni (Figure 5), suggests a unique host-interaction with lysine catabolism in the early evolution of *Wolbachia* in plant-feeding hosts. Given the evidence that lysine is often limiting in plants and plant roots (Galili, 2002; Pratelli and Pilot, 2014; Galili et al., 2016) it is curious that the eukaryote-like *sdh* gene has been conserved. The *sdh* gene is rare in prokaryotes not living under high osmotic stress (Neshich et al., 2013), so the presence of this gene is unexpected. In *Caenorhabditis elegans*, saccharopine excess was found to be toxic to mitochondria (Zhou et al., 2019), so it is possible that SDH in these *Wolbachia* acts to reduce saccharopine toxicity intracellularly. But it would seem unlikely that lysine excess leading to saccharopine excess would be present intracellularly in PPNs if lysine is already limiting in their diets. However, saccharopine excess might arise if there is downregulation of lysine catabolism in roots in response to nematode infection, as has been proposed previously (Pratelli and Pilot, 2014). Alternatively, we suggest the *sdh*-HGT in these *Wolbachia* may function to mediate nematode triggering of plant systemic acquired resistance (SAR) through the lysine-to-pipecolic acid (Lys-Pip) system (Návarová et al., 2013; Yang and Ludewig, 2014). Our model for this interaction derives from two sources. First, evidence suggests that plant-parasitic nematodes can induce plant amino acid importers in root cells including amino acid permeases (AAPs AtAAP1-8), lysine/histidine transporters (LHT), and cationic amino acid transporters (CAT AtCAT6) (Hammes et al., 2006; Elashry et al., 2013; Marella et al., 2013), hijacking the existing plant system to acquire limiting aspartate-derived amino acids such as lysine (Yang and Ludewig, 2014). Second, evidence suggests that plants specifically mediate free soluble lysine upon bacterial infection of leaves, first increasing lysine import then massively upregulating lysine catabolism to produce an excess of pipecolic acid (Pip), which then acts as the major metabolic regulator/intensifier of SAR defense (Yang and Ludewig, 2014; Yang et al., 2014) and downregulating lysine synthesis in leaves, reducing this limiting amino acid in roots. Both SAR and decreased lysine will be unfavorable for PPNs and plant pests, thus dysregulating the host-plant Lys-Pip system *via* secreted SDH from this eukaryote-derived *sdh*-HGT could be favorable. Further experiments will be needed to assess this hypothesis directly.

*Wolbachia* genomes from PPNs were also enriched for several other nutrient biosynthesis GO terms including heme and protoporphyrinogen IX (PPG) (part of the heme synthesis pathway). Consistent with gene repertoire enrichment on heme-related pathways, selection analysis examining dN/dS indicated high purifying selection on PPG biosynthesis in PPN-type *Wolbachia* compared to other *Wolbachia*. Furthermore, selection analyses also suggested heme-binding was under strong purifying selection in pairwise comparisons between strains *w*Tex and *w*Ppe. Together, these findings are consistent with the ‘iron hypothesis’ for *Wolbachia* which posits that heme biosynthesis and iron homeostasis may be central to the maintenance of *Wolbachia* (Foster et al., 2005; Wu et al., 2009; Darby et al., 2012; Gill et al., 2014; Luck et al., 2014; Brown et al., 2016). Nematodes are exceptional among animals in having lost the ability to synthesize heme early in their evolution as bacterivores (Rao et al., 2005; Slatko et al., 2010; Elsworth et al., 2011; Kořený et al., 2021). Most nematodes, as bacterivores, can extract ample heme from their diets. However, descendants of the early bacterivore nematodes such as PPNs and filarial nematodes that evolved to specialize in non-bacterial diets will have had access to limited heme. These PPNs and filarial nematodes are, then, perhaps not surprisingly the only groups to host *Wolbachia* symbionts. Consistent with the struggle to regain heme in heme-depleted diets, many nematodes have gained a functional HGT of an ancient alphaproteobacterial ferrochelatase gene, the last step in heme synthesis (Wu et al., 2013). Considering our findings, which suggest enhanced essentiality and conservation of heme/PPG in PPN-type *Wolbachia*, we suggest these *Wolbachia* heme pathways may have been pivotal in the transition of nematodes to the plant-parasitic lifestyle and may explain the apparent persistence of *Wolbachia* in certain nematode clades, but not widely across others. It is not clear why genes for PPG biosynthesis would be enriched in PPN-type *Wolbachia* compared to other groups, including filarial nematodes. However, one possibility is that these PPN *Wolbachia* strains may generate excess protoporphyrin as a toxin. The observation that *Wolbachia w*Ppe is localized adjacent to the esophageal glands (Brown et al., 2016) could indicate a role for these *Wolbachia* in producing protoporphyrin destined for nematode secretions during migratory endoparasitic feeding, which may trigger programmed cell death in plant roots by disrupting mitochondrial membranes (Kořený et al., 2021), facilitating nematode feeding.

*Wolbachia* genomes from PPNs were also enriched for thiamine (vitamin B1) and fatty acid biosynthesis compared to genomes from other *Wolbachia*. Strain *w*Tex was further enriched for thiamine and thiamine diphosphate biosynthesis genes compared to strain *w*Ppe. This enrichment derived from a multi-gene thiamine synthesis operon (*thiE-thiM-thiD*) that was only shared with the cat flea *Wolbachia w*CfeT, but no other *Wolbachia*, representing a likely HGT from spirochetes. This additional operon suggests some additional thiamine needs in *w*Tex. While thiamine synthesis genes (*iscS*/*adk*) are universal in *Wolbachia*, others, like *tenA* occur only in a few strains (Lefoulon et al., 2020), whereas *Wolbachia* in blood-feeding hosts may have acquired genes for thiamine salvage (Nikoh et al., 2014). Thiamine biosynthesis enrichment in PPN-type *Wolbachia* may derive from a need to supplement this limited essential vitamin, which, like the amino acid lysine, is largely restricted to chloroplast-dense leaves and may be depleted in roots (Martinis et al., 2016). Interestingly, we found in the transition from *Wolbachia* from *w*Fol from springtails to *w*CfeT from cat fleas, there appeared to be higher than expected dN/dS on thiamine pyrophosphate binding, which is involved in thiamine transport, suggesting a possible change in thiamine needs in these basal branches of *Wolbachia*. Conversely, interpreting the enriched fatty acid biosynthesis in PPN-type *Wolbachia* is more difficult; there are no known fatty acids that are essential (not synthesized) by nematodes (Zečić et al., 2019). However, fatty acids are likely absorbed by nematodes and their uptake could be variable among parasitic nematodes (Mondal et al., 2016) depending on the availability in roots, or potentially through supply by *Wolbachia*.

To investigate possible functions linked to *Wolbachia*’s success in early-branching clades, we analyzed other patterns in purifying selection indicated by measures of dN/dS. As might be expected, we found universally high purifying selection on housekeeping activities such as ribonucleotide binding, DNA topoisomerase type II, and DNA topological change, but early-branching *Wolbachia* also showed enhanced purifying selection for energy and respiration-related activities including ATP binding, NADH dehydrogenase activity, quinone binding, and respirasome activity. These conserved respiration functions may relate to host mitochondria-*Wolbachia* interaction homeostasis, which has been shown as critical to maintenance in host cells – and disruption of oxidative phosphorylation leading to host cell death in alternate hosts (Uribe-Alvarez et al., 2019). Among *w*Tex and *w*Ppe, there appeared to be the highest purifying selection on metabolic functions associated with energy, and metal ion, iron-sulfur cluster, heme binding, which again point to oxygenic-mitochondrial and heme synthesis processes and mitochondria-*Wolbachia* interaction and homeostasis as key functions specifically conserved in PPN *Wolbachia*. Conversely, results showed lower purifying selection – or potentially, directional selection – within *w*Tex and *w*Ppe for various nutrient metabolism processes including arginine metabolism, lysine biosynthesis, and binding of vitamin B6, heme, and zinc ions, as well as protein folding and transport, chaperone binding, and aromatic amino acid synthesis, suggesting these processes, which likely influence host-*Wolbachia* interactions, are uniquely important in supergroup L *Wolbachia*. Arginine biosynthesis, however, was under higher purifying selection in the subsequent branch between PPN-type *Wolbachia* and strain *w*Pni, as processed including protein transport by the Tat complex and proteolysis, suggesting these are core, conserved functions in these early-branching *Wolbachia* clades. Conversely, higher dN/dS pathways between supergroup L and *w*Pni suggested evolutionary changes in amino ugar metabolism and many cell shape/division functions in these early *Wolbachia* host transitions.

While *Wolbachia*-like gene transfers to their eukaryote hosts have been reported in numerous studies (McNulty et al., 2010; Dunning Hotopp, 2011; Brelsfoard et al., 2014; Koutsovoulos et al., 2014; Husnik and McCutcheon, 2018), there has been limited study of HGTs to *Wolbachia* from other microbes. Our results here suggest some of these hypothesized HGTs may be important. For example, in addition to the putative HGTs for lysine synthesis (*asd2-lysC*), *sdh*, and thiamine synthesis (*thiE-thiM-thiD*), which dN/dS patterns suggest are under enhanced purifying selection, we found other conserved putative HGTs of interest. For example, both PPN *Wolbachia* shared a predicted large HGT from *Rickettsia* for the gene *tlcA*, which is critically important for parasitism-related ATP import or exchange in *Rickettsia* (Audia and Winkler, 2006; Renvoisé et al., 2011). This *tlcA*-HGT is curiously absent in other *Wolbachia* and Anaplasmataceae. There was also a squalene/phytoene synthase gene of putative HGT origin, with the closest match to carotenoid synthesis-associated genes from the conifer aphid *C. cedri* (Nováková and Moran, 2012), adjacent to *dxr*, which is an essential gene in the MEP pathway of isoprenoid synthesis and occurs across *Wolbachia*. It is unclear how these genes, or others such as the eukaryote-like PCBD1-HGT which may act in phenylalanine metabolism to tyrosine, function in PPN *Wolbachia*. In the future, we suggest the systematic study of such genes would be warranted. To improve such studies, efforts should focus on overcoming genome completeness for MAG datasets. While our *w*Tex genome may be incomplete, due to low coverage and variance among strains from pooled hosts, we expect most of our findings and conclusions discussed here are conservative in that the essential and shared genes were likely detected through our assembly method that centered on extracting contigs with blastn matches to *w*Ppe, *Wolbachia*, and outgroups. In contrast, we predict that the genes most likely to be missed in our *w*Tex assembly by these methods would be those that are most diverged and regions with novel HGTs that do not map to other *Wolbachia*. Future work should focus on improving coverage and read length to overcome these issues.

Besides function, our study sought to investigate the distribution of PPN *Wolbachia*. *Wolbachia w*Tex was found with shallow read depth within these nematode community assemblies, however, relative to the predicted nematode host which had COI gene coverage of ∼2–8× per sample, it had similar coverage per sample (∼0.7–8×) which suggests a high titer in its hosts, based on an estimation of ∼300 mitochondria per host cell. Furthermore, although *Wolbachia*-like sequence reads were obtained in 10/16 of our sampled sites, only one site had sequences matching PPN-type *Wolbachia*, despite the presence of hundreds of different nematodes in these samples, based on COI profiling. However, for the *Wolbachia*-positive site, which was a tropical fruit farm in southern Texas, *w*Tex was in nematode communities from several plants, suggesting its host is not specific to plant species. The possible host of *w*Tex may be the spiral nematode, *Helicotylenchus* sp., based on abundance correlation analysis. *Helicotylenchus* spp. are sometimes ectoparasitic, but some species are migratory and burrowing (e.g., the banana spiral nematode *Helicotylenchus multicinctus*), a lifestyle resembling hosts of other PPN *Wolbachia*, *P. penetrans*, and *R. similis*. This scenario puts a spotlight on future studies of how *Wolbachia* may play a role-specific to this migratory endoparasitic lifestyle.

Screening of SRA data revealed potential PPN *Wolbachia* in global soils and rhizospheres. We found the prevalence at about 0.42% of samples, but with few reads per run, suggesting these *Wolbachia* occur at low titers, at least at the bulk community level. However, these data likely under represent PPN *Wolbachia* diversity, prevalence, and titer because nematode distribution is patchy, with typical soil samples (∼0.25 g) not capturing much diversity (Donn et al., 2008) and common soil DNA isolation practices often failing to break nematode cuticles (Waeyenberge et al., 2019; Sloan et al., 2021). Universal primer-based amplicon sequencing for PPN *Wolbachia* is an improvement over PCR methods because most *Wolbachia*-specific 16S PCR primers have significant mismatches to these early-branching strains (e.g., primers Wolb-SpecF and SpecR and Wol-F-1992 and Wol-R-1992; and the primers Wol_16S_F and Wol_281B_F which match *w*Ppe have mismatches to *w*Tex). However, the major limitation of amplicon data mining is the short length of amplicon sequencing reads which limits the information gained, compared to more costly WGS methods.

## Conclusion

This study expands our understanding of early-branching *Wolbachia*, pointing to unique genes and pathways that give insights into the functions of these elusive PPN *Wolbachia* strains. Examples include conserved putative HGTs for lysine, thiamine, and heme/protoporphyrinogen IX biosynthesis and genes that may interact with plant immunity, and other enriched pathways with distinct signatures of selection. These enrichment analyses add to the tool set that should be useful for future studies on new *Wolbachia*. Our community WGS and SRA screens illuminate the broad global and phylogenetic distribution of PPN-type *Wolbachia*. One major focus for future study of these early-branching *Wolbachia* will be to investigate the ∼40% of predicted genes that were recovered with no match to genes with known function. Other key questions that require further work are the fitness effects of these *Wolbachia* on their hosts, which will require improved lab culturing of the nematodes.

## Data Availability Statement

The datasets presented in this study can be found in online repositories. The names of the repository/repositories and accession number(s) can be found below: https://www.ncbi.nlm.nih.gov/genbank/, JAIXMJ000000000 and https://www.ncbi.nlm.nih.gov/, PRJNA687334.

## Author Contributions

NW and SA assisted with nematode isolation, PCR, and genomic library preparation. AB led the design of experiments, developed bioinformatics code, and pipelines, and drafted the manuscript. All co-authors assisted with the revision of the final manuscript.

## Conflict of Interest

The authors declare that the research was conducted in the absence of any commercial or financial relationships that could be construed as a potential conflict of interest.

## Publisher’s Note

All claims expressed in this article are solely those of the authors and do not necessarily represent those of their affiliated organizations, or those of the publisher, the editors and the reviewers. Any product that may be evaluated in this article, or claim that may be made by its manufacturer, is not guaranteed or endorsed by the publisher.

## Footnotes

1
https://github.com/tseemann/barrnap

2
https://github.com/rambaut/figtree/releases

3
https://github.com/lyijin/topGO_pipeline/
