# Supplementary material for: Discovery of Early-Branching Wolbachia Reveals Functional Enrichment on Horizontally Transferred Genes
Source: Front Microbiol. 2022 Apr 25;13:867392. doi: 10.3389/fmicb.2022.867392 (PMC9084900; doi:10.3389/fmicb.2022.867392)
Supplement: Supplementary file 1 [file Data_Sheet_1.docx]

Supplementary Material

**Supplementary Table 1**: Collection data for nematode communities screened for plant-parasitic nematode-associated *Wolbachia*-like 16S rRNA gene regions. Samples with * indicate positive presence of plant-parasitic nematode-type *Wolbachia* matches.

| Sample Name | Host Plant(s) | Geographic Location & Habitat | Latitude/ Longitude | NCBI Sample Accession |
| --- | --- | --- | --- | --- |
| Avocado* | Avocado tree (*Persea americana*) | Los Fresnos, TX (fruit farm) | 26.158570 / -97.384450 | SAMN17141360 |
| Sugar* | Sugar-apple tree (*Annona squamosa*) | Los Fresnos, TX (fruit farm) | 26.158570 / -97.384450 | SAMN17141361 |
| Plantain* | Plantain (*Musa*×*paradisiaca*) | Los Fresnos, TX (fruit farm) | 26.158570 / -97.384450 | SAMN17141359 |
| P3-11* | Various fruit trees | Los Fresnos, TX (fruit farm) | 26.158570 / -97.384450 | SAMN17373384 |
| Guava* | Guava tree (*Psidium guajava*) | Los Fresnos, TX (fruit farm) | 26.158570 / -97.384450 | SAMN17141358 |
| Rosetta* | Rosetta apple (*Malus pumila*) | Los Fresnos, TX (fruit farm) | 26.158570 / -97.384450 | SAMN17141357 |
| P15 | Citrus trees | Los Fresnos, TX (fruit farm) | 26.132554 / -97.473898 | SAMN17373379 |
| P18 | Mixed grasses | Los Fresnos, TX (wild plants at roadside) | 26.125003 / -97.395915 | SAMN17373380 |
| P19 | Mixed grasses | La Pryor, TX (wild) | 29.662474 / -95.376874 | SAMN17141341 |
| P21 | Mixed grasses | Junction, TX | 30.479468 / -99.778204 | SAMN17373381 |
| P22 | Mixed shrubs | Carlsbad, NM (wild) | 32.438692 / -104.178553 | SAMN17373382 |
| P23 | Mixed shrubs | Laredo, TX (wild) | 27.535932 / -99.433883 | SAMN17373383 |
| P24 | Olive tree (*Olea europaea*) | Weslaco, TX (research farm) | 26.167820 / -98.010570 | SAMN17141346 |
| P29 | Mixed vegetables | Lubbock, TX (community farm) | 33.517183 / -101.811984 | SAMN17141350 |
| P12 | Coffee (*Coffea arabica*) | Weslaco, TX (research farm) | 26.17292 / -79.99893 | SAMN17141337 |
| P31 | Mixed grass roots | Lampasas, TX (wild) | 30.962257 / -98.052659 | SAMN17141354 |
| P32 | Mixed grass soil | Lampasas, TX (wild) | 30.962257 / -98.052659 | SAMN17141355 |
| P2 | Mixed shrubs/grasses | Lubbock, TX (meadow adjacent to golf course) | 33.59638 / -101.94378 | SAMN17141351 |
| P20 | Mixed shrubs/grasses | Carrizo Springs, TX (wild) | 28.51244 / -99.87207 | SAMN17141342 |
| P26 | Mixed grasses (soil) | Corvallis, OR (wild, at creek bed) | 44.60424/ -123.21597 | SAMN17141347 |
| P27 | Mixed grasses (roots only) | Corvallis, OR (wild, at creek bed) | 44.60424/ -123.21597 | SAMN17141348 |

**Supplementary Methods: Draft Genome Assembly Details**

To recover nematode and symbiont genomes, reads were *de novo* assembled, then *Wolbachia*-like contigs were annotated. First, reads for each sample were filtered and trimmed using Trimmomatic v.0.38 (Bolger et al., 2014) and overlaps in paired reads were detected and merged in Pear v0.9.11 (Zhang et al., 2014). Filtered paired reads and merged reads were *de novo* assembled with metaSPAdes v.3.13.0 (Bankevich et al., 2012; Nurk et al., 2017) using low kmers (25, 33, 45). Initial assembly quality assessment was performed using Quast v5.0.1 (Gurevich et al., 2013). As part of a survey, assemblies were screened for *Wolbachia*-like 16S rRNA using a two-step analysis with blastn in Blast+ v2.10.1 (Camacho et al., 2009) (-evalue 10) first to a custom database of *Wolbachia* 16S rRNA sequences, and then a second blastn to the complete NCBI nt database. Samples with top blast hits matching *Wolbachia* strains from plant-parasitic nematodes (*w*Ppe and *w*Rad) more closely than other non-plant parasitic nematode strains were considered “positive” for PPN *Wolbachia*. Furthermore, to increase screening sensitivity for low-level infections in the pooled nematode samples, we also performed the same two-step blastn analysis for raw and filtered reads, anticipating that low-level *Wolbachia* infections may fail to assemble well in metaSPAdes. Any samples with top blastn hits to PPN *Wolbachia* from this read-based blast were also considered “positive”. For PPN *Wolbachia*-positive samples, full genomes were extracted using similar two-step blastn searches, first to *Wolbachia* genome databases, then to the full nt database.

Due to the low coverage and incompleteness of the initial *Wolbachia* genomes at this stage, we examined sequence similarity among assembled contigs from positive samples. Based on the high sequence similarity among samples, and their origin from the same farm, we combined these samples for further analysis, to improve coverage and assembly quality. However, the combined read data was close to 1 TB in size and therefore not practical to assemble using metaSPAdes due to RAM limitations, so instead, we used an iterative map-assemble approach using bwa v.0.1.17 (Li and Durbin, 2009). Because initial sequence similarity analysis suggested the new *Wolbachia* isolate was significantly diverged from previous *Wolbachia* strains, the bwa mem approach resulted in poor retrieval of reads, and we anticipated this would be particularly problematic for recovering divergent regions and accessory genes not present in the reference *Wolbachia* strains. Therefore, we instead used a subtractive mapping approach as follows. First, we identified non-*Wolbachia* contigs in the initial assembles using both blastn results and a %GC filter using prinseq-lite.pl in the BRAbB software (Brankovics et al., 2016), then we used bwa mem to map each sample’s reads to this non-*Wolbachia* data specific to our samples, then we used samtools v.1.9 (Li et al., 2009) and custom scripts to extract unmapped (i.e. *Wolbachia*-enriched) reads. These enriched reads were concatenated for all samples, and assembled in metaSPAdes with a kmers (25, 45, 65, 99). Resulting assemblies were examined again with a two-step blastn against the earlier *Wolbachia* genome database combined with *Wolbachia*-like contigs from the individual samples with the new strain. The process of bwa-based subtractive mapping was repeated from the original reads with this new, improved database. The new *Wolbachia*-enriched reads were again concatenated and *de novo* assembled again with metaSPAdes. This approach was repeated three times, and stopped once the sum of the length of the resulting new strain *Wolbachia*-like contigs ceased to increase between cycles and inspecting genome contamination and completeness metrics using CheckM v1.0.18 (Parks et al., 2015) at intermediate steps.

Resulting contigs were assessed by several quality controls to reduce the likelihood of spurious bioinformatic contamination with non-*Wolbachia* data. First, blastn to the nt database was used to check that each final contig’s highest blast hit was to *Wolbachia* or its closest outgroup Rickettsiales (*Ehrlichia*, *Neoehrlichia*, *Anaplasma*). Next, to filter out contigs with potential short horizontally transferred *Wolbachia*-like DNA regions (HGTs), long contigs (>5,000 bp) were removed if coverage was >2 times the average coverage of the longest contigs, using coverage analysis in pileup.sh in BBMap v.38.90 (Bushnell, 2014). Similarly, to filter out possible HGTs, contigs were imported into Geneious Prime v2020.0.4 (Biomatters, Ltd) and inspected, with contigs >1,000 bp removed if GC content was below 24% or above 42%. Next, quality was assessed by annotating contigs using Prokka v.1.14.6 (Seemann et al., 2014) which uses Prodigal for *ab initio* gene prediction, HMMER3 for protein family profiles, BLAST+ for comparative annotation, Barrnap (<https://github.com/tseemann/barrnap>) for rRNAs, Aragorn (Laslett and Canback, 2004) for tRNAs. Resulting genes were then analyzed with blastn to the nt database and with DIAMOND blastx to the full diamond-formatted nr database. If contigs contained genes with higher similarity to outgroup Rickettsiales, or to non-PPN *Wolbachia*, rather than to PPN *Wolbachia*, they were removed unless the same contigs contained more genes with highest similarity to PPN *Wolbachia*. Finally, to check for regions of possible bifurcating misassembly due to mutational differences in the field sampled specimens, contigs were aligned and checked for blocks of near identity and synteny using Geneious Prime plugins ProgressiveMauve v1.1.1 (Darling et al., 2010) and LASTZ alignment v7.0.2 (Biomatters, Ltd). This resulted in joining of several overlapping and removal of several suspected regions of bifurcating misassembly leading to false duplicates.

**References Cited**

Bankevich, A., Nurk, S., Antipov, D., Gurevich, A. A., Dvorkin, M., Kulikov, A. S., et al. (2012). SPAdes: A new genome assembly algorithm and its applications to single-cell sequencing. *J. Comput. Biol.* 19, 455–477. doi:10.1089/cmb.2012.0021.

Bolger, A. M., Lohse, M., and Usadel, B. (2014). Trimmomatic: A flexible trimmer for Illumina sequence data. *Bioinformatics* 30, 2114–2120. doi:10.1093/bioinformatics/btu170.

Brankovics, B., Zhang, H., van Diepeningen, A. D., van der Lee, T. A. J., Waalwijk, C., and de Hoog, G. S. (2016). GRAbB: Selective Assembly of Genomic Regions, a New Niche for Genomic Research. *PLoS Comput. Biol.* 12, 1–9. doi:10.1371/journal.pcbi.1004753.

Bushnell, B. (2014). BBMap: A fast, accurate, splice-aware aligner. *No. LBNL-7065E. . Lawrence Berkeley Natl. Lab.(LBNL), Berkeley, CA (United States)*.

Camacho, C., Coulouris, G., Avagyan, V., Ma, N., Papadopoulos, J., Bealer, K., et al. (2009). BLAST+: Architecture and applications. *BMC Bioinformatics* 10, 1–9. doi:10.1186/1471-2105-10-421.

Darling, A. E., Mau, B., and Perna, N. T. (2010). Progressivemauve: Multiple genome alignment with gene gain, loss and rearrangement. *PLoS One* 5, e11147. doi:10.1371/journal.pone.0011147.

Gurevich, A., Saveliev, V., Vyahhi, N., and Tesler, G. (2013). QUAST: Quality assessment tool for genome assemblies. *Bioinformatics* 29, 1072–1075. doi:10.1093/bioinformatics/btt086.

Laslett, D., and Canback, B. (2004). ARAGORN, a program to detect tRNA genes and tmRNA genes in nucleotide sequences. *Nucleic Acids Res.* 32, 11–16. doi:10.1093/nar/gkh152.

Li, H., and Durbin, R. (2009). Fast and accurate short read alignment with Burrows-Wheeler transform. *Bioinformatics* 25, 1754–1760. doi:10.1093/bioinformatics/btp324.

Li, H., Handsaker, B., Wysoker, A., Fennell, T., Ruan, J., Homer, N., et al. (2009). The Sequence Alignment/Map format and SAMtools. *Bioinformatics* 25, 2078–2079. doi:10.1093/bioinformatics/btp352.

Nurk, S., Meleshko, D., Korobeynikov, A., and Pevzner, P. A. (2017). MetaSPAdes: A new versatile metagenomic assembler. *Genome Res.* 27, 824–834. doi:10.1101/gr.213959.116.

Parks, D. H., Imelfort, M., Skennerton, C. T., Hugenholtz, P., and Tyson, G. W. (2015). CheckM: Assessing the quality of microbial genomes recovered from isolates, single cells, and metagenomes. *Genome Res.* 25, 1043–1055. doi:10.1101/gr.186072.114.

Zhang, J., Kobert, K., Flouri, T., and Stamatakis, A. (2014). PEAR: A fast and accurate Illumina Paired-End reAd mergeR. *Bioinformatics* 30, 614–620. doi:10.1093/bioinformatics/btt593.

**Supplementary Table 2**: Sequence reads and assembly statistics for *Wolbachia* from *Wolbachia*-positive nematode samples.

| Sample Name | Total  Reads  (millions) | Filtered & Merged Reads  (millions) | Reads Mapped to Final *Wolbachia*  Assembly  (millions) | *Wolbachia* Scaffolds | Maximum *Wolbachia* Scaffold Length | *Wolbachia* Contig  N50 | *Wolbachia* Assembly Coverage |
| --- | --- | --- | --- | --- | --- | --- | --- |
| Avocado | 780.10 | 336.79 | 18.39 | 283 | 2261 | 1769 | 3.93 |
| Sugar | 662.47 | 367.36 | 17.55 | 378 | 1968 | 720 | 4.30 |
| Plantain | 243.27 | 146.50 | 5.98 | 353 | 4346 | 1247 | 5.93 |
| P3-11 | 704.10 | 436.30 | 6.63 | 272 | 3706 | 860 | 8.08 |
| Guava | 247.75 | 102.67 | 6.89 | 197 | 837 | 681 | 3.61 |
| Rosetta | 31.65 | 20.88 | 0.70 | 2 | 753 | 753 | 0.69 |
| Combined Sample  Iterative Assembly | 2,670.09 | 1,410.5 | 56.14 | 192 | 57,862 | 10,082 | 15.96 |

**Supplementary Figure 1**: Spearman correlation heatmap among nematode community members and *Wolbachia* strain *w*Tex, based on coverage of top cytochrome oxidase (COI) gene coverage for nematodes. Spearman’s rho R-values are depicted with blue/orange shading and uncorrected statistically significant p-values <0.05 are shown with red asterisks. After multiple testing correction using the Benjamini & Hochberg (1995) method, *w*Tex (in black boxes) showed significant correlation only with two nematode sequence groups, with only one (Heteroderidae sp. CD2526, matching *Helicotylenchus* and *Rotylenchus*, shown in bold) assembling as long scaffolds. Black star marks the intersection of *w*Tex with this Heteroderidae sp. CD2526 match.

**Supplementary Figure 2.** Phylogeny of sequences with blastn matches to the nematode cytochrome oxidase 1 (COI) gene that were significantly correlated with *Wolbachia*-positive samples based on 1,266 aligned positions of the partial COI gene and adjacent regions from 6 samples in this study compared with sequences from GenBank. Maximum likelihood phylogeny reconstruction was performed in RAxML GTR+Gamma with bootstrap support from 500 replicates is shown on branches. Most supported nodes were obtained with high support in Bayesian 50% majority rule in MrBayes with GTR+G with 4 rate categories model. Bayesian posterior probabilities are shown in bold after bootstrap values on branches. Sequences obtained in this study are indicated in bold blue font. Blue and yellow highlights show groups with previously sequenced *Wolbachia* strains, and groups with green and pink highlights show clades possibly associated with *w*Tex.


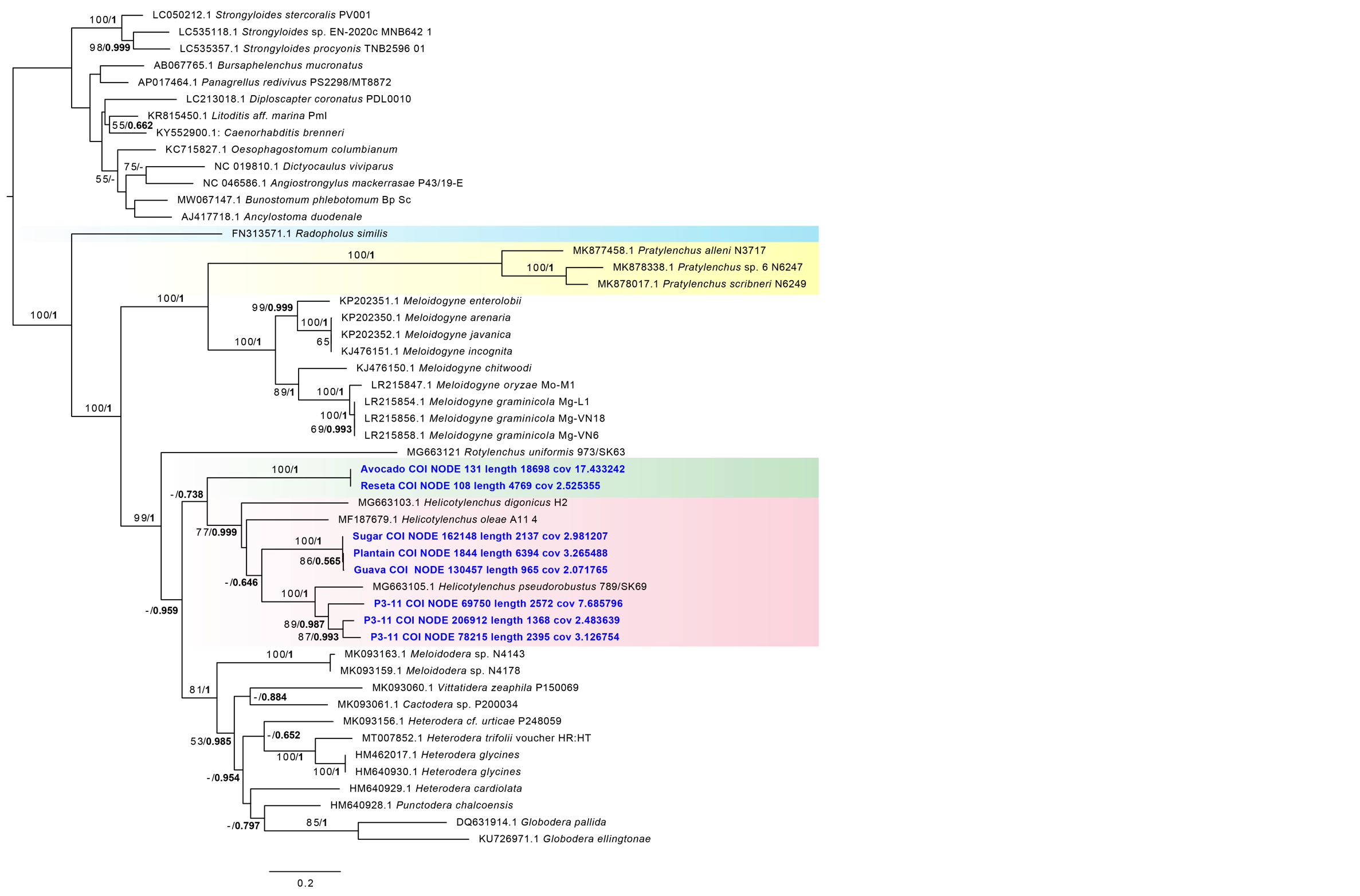


**Supplementary Figure 3.** Phylogeny of sequences with blastn matches to the nematode cytochrome oxidase 1 (COI) gene that were significantly correlated with *Wolbachia*-positive samples based on 472 aligned positions of the partial COI gene and adjacent regions from 6 samples in this study compared with sequences from GenBank. This analysis is similar to that shown in **Supplementary Figure 2** except that it includes a shorter region of the COI gene to include more isolates. Maximum likelihood phylogeny reconstruction was performed in RAxML GTR+Gamma with bootstrap support from 500 replicates is shown on branches. Most supported nodes were obtained with high support in Bayesian 50% majority rule in MrBayes with GTR+G with 4 rate categories model. Bayesian posterior probabilities are shown in bold after bootstrap values on branches. Sequences obtained in this study are indicated in bold blue font. Blue and yellow highlights show groups with previously sequenced *Wolbachia* strains, and groups with green and pink highlights show clades possibly associated with *w*Tex.


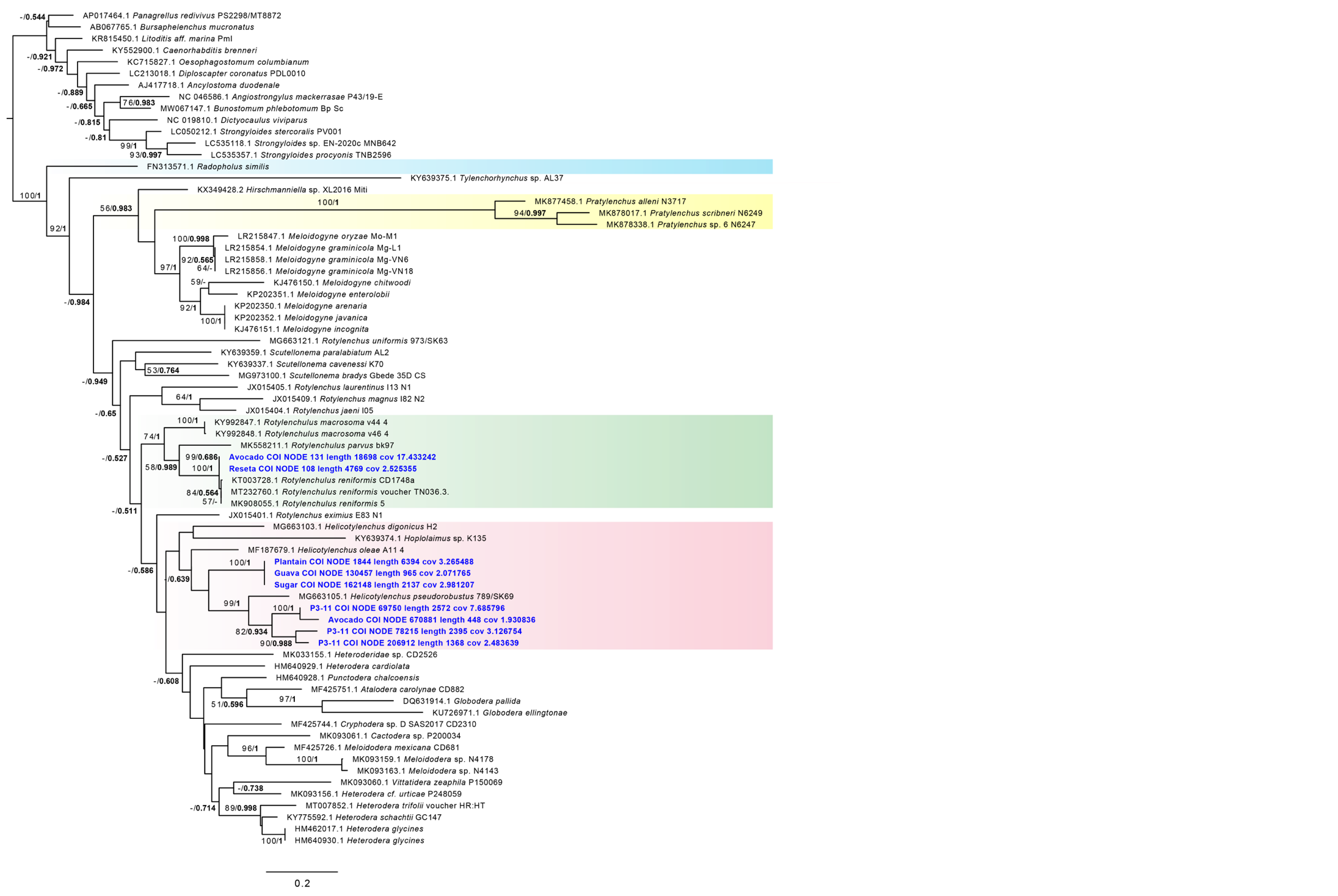


**Supplementary Figure 4.** Phylogeny of the partial 16S rRNA gene for *Wolbachia* and outgroups based on 1,573 aligned positions. Maximum likelihood phylogeny reconstruction was performed in RAxML GTR+Gamma with bootstrap support >50% from 1000 replicates shown on branches. Most supported nodes were obtained with high support in Bayesian 50% majority rule in MrBayes with GTR+G with 4 rate categories model. Sequences obtained in this study are indicated in bold orange font. Strains clustering adjacent to supergroup M are shown in bold black font.

**
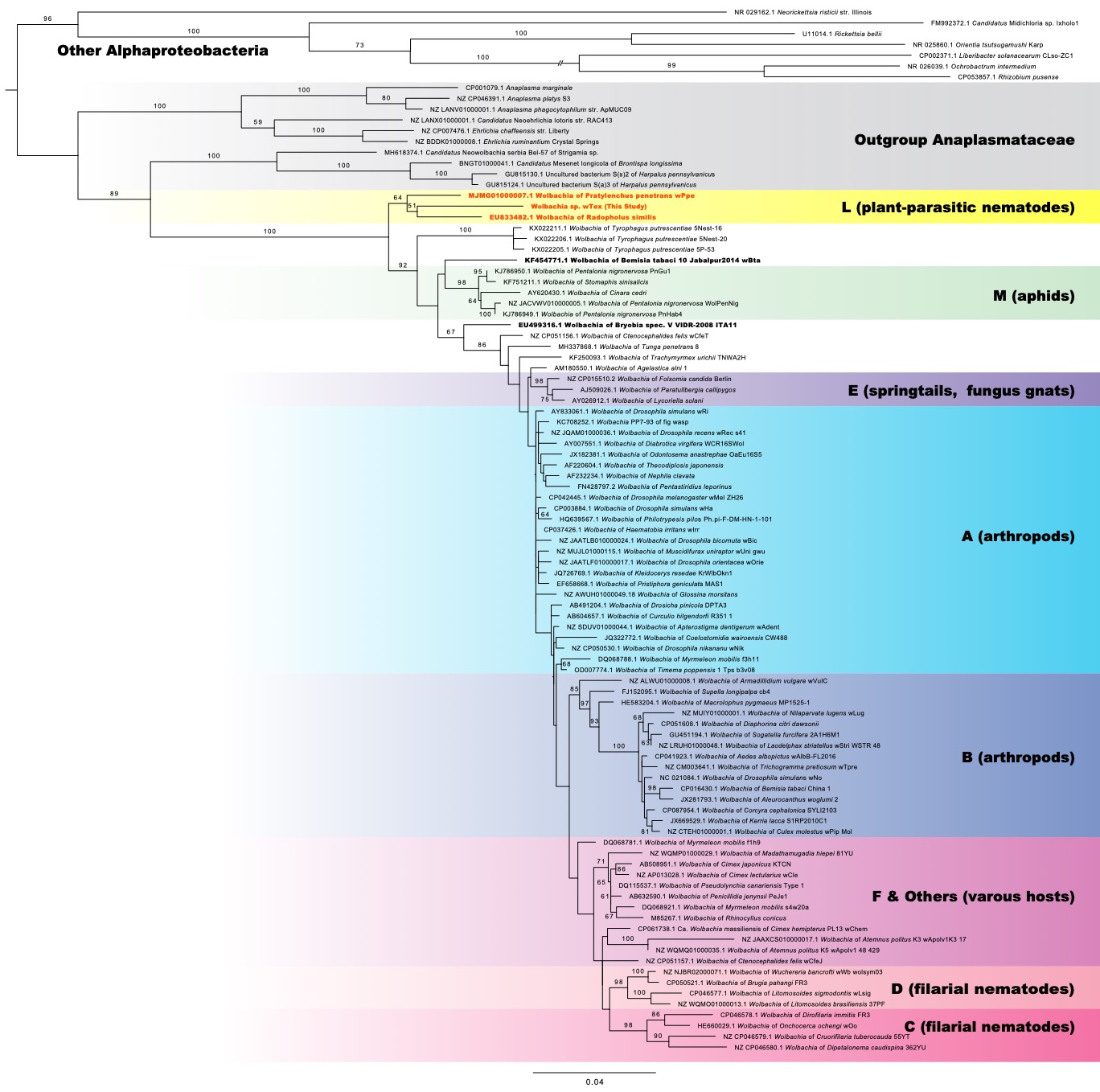
**

**Supplementary Figure 5**. Phylogeny of plant-parasitic nematode-type *Wolbachia*-like matches from SRA database hits for the 0-550 region of the 16S rRNA gene, based on 1,536 aligned positions. Maximum likelihood phylogeny reconstruction was performed in RAxML GTR+Gamma. Bootstrap support >50% from 500 replicates is shown on branches along with posterior probabilities from Bayesian 50% majority rule in MrBayes with GTR+G with 4 rate categories model, shown only for nodes close to *Wolbachia* *w*Tex or other relevant nodes for SRA hits. Sequences from the SRA are indicated in bold blue font and sequences from PPN *Wolbachia* are indicated in orange bold font. Highlights show clades with *Wolbachia*-like hits.


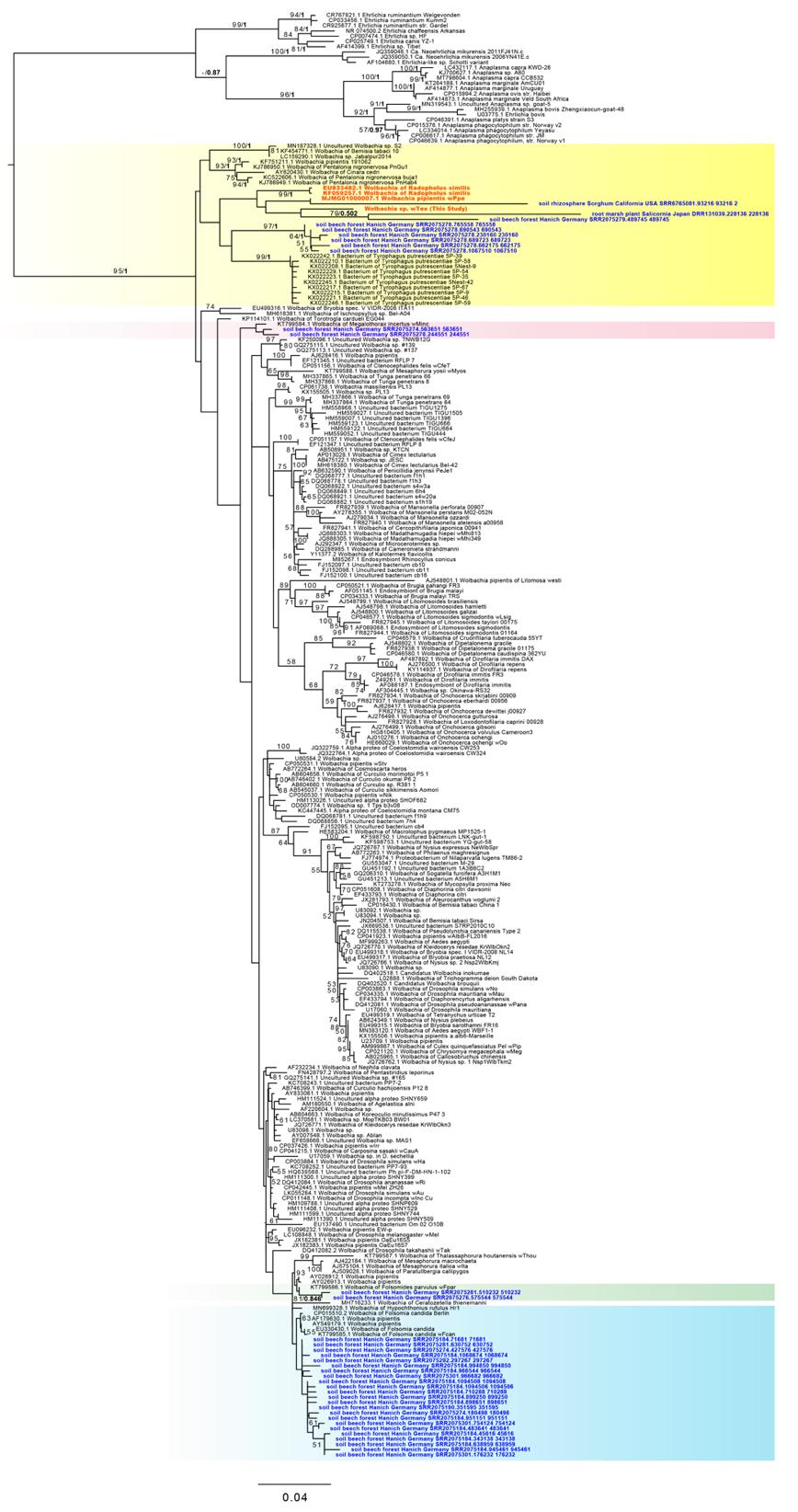


**Supplementary Figure 6**. Phylogeny of plant-parasitic nematode-type *Wolbachia*-like matches from SRA database hits for the 490-800 region of the 16S rRNA gene, based on 1,536 aligned positions. Maximum likelihood phylogeny reconstruction was performed in RAxML GTR+Gamma. Bootstrap support >50% from 500 replicates is shown on branches along with posterior probabilities from Bayesian 50% majority rule in MrBayes with GTR+G with 4 rate categories model, shown only for nodes close to *Wolbachia* *w*Tex or other relevant nodes for SRA hits. Sequences from the SRA are indicated in bold blue font and sequences from PPN *Wolbachia* are indicated in orange bold font. Highlights show clades with *Wolbachia*-like hits.


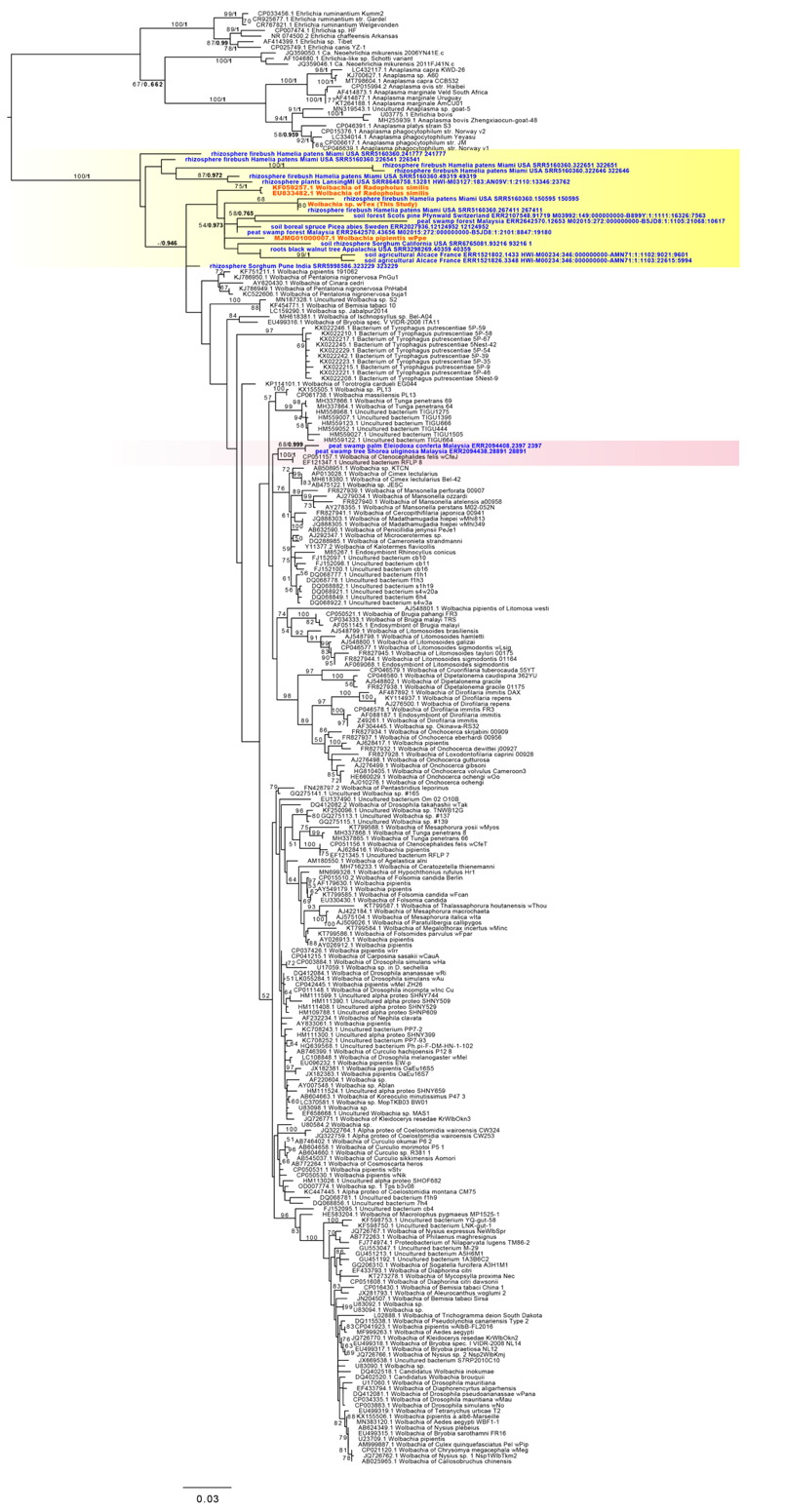


**Supplementary Figure 7**. Phylogeny of plant-parasitic nematode-type *Wolbachia*-like matches from SRA database hits for the 760-1050 region of the 16S rRNA gene, based on 1,536 aligned positions. Maximum likelihood phylogeny reconstruction was performed in RAxML GTR+Gamma. Bootstrap support >50% from 500 replicates is shown on branches along with posterior probabilities from Bayesian 50% majority rule in MrBayes with GTR+G with 4 rate categories model, shown only for nodes close to *Wolbachia* *w*Tex or other relevant nodes for SRA hits. Sequences from the SRA are indicated in bold blue font and sequences from PPN *Wolbachia* are indicated in orange bold font. Highlights show clades with *Wolbachia*-like hits.

**
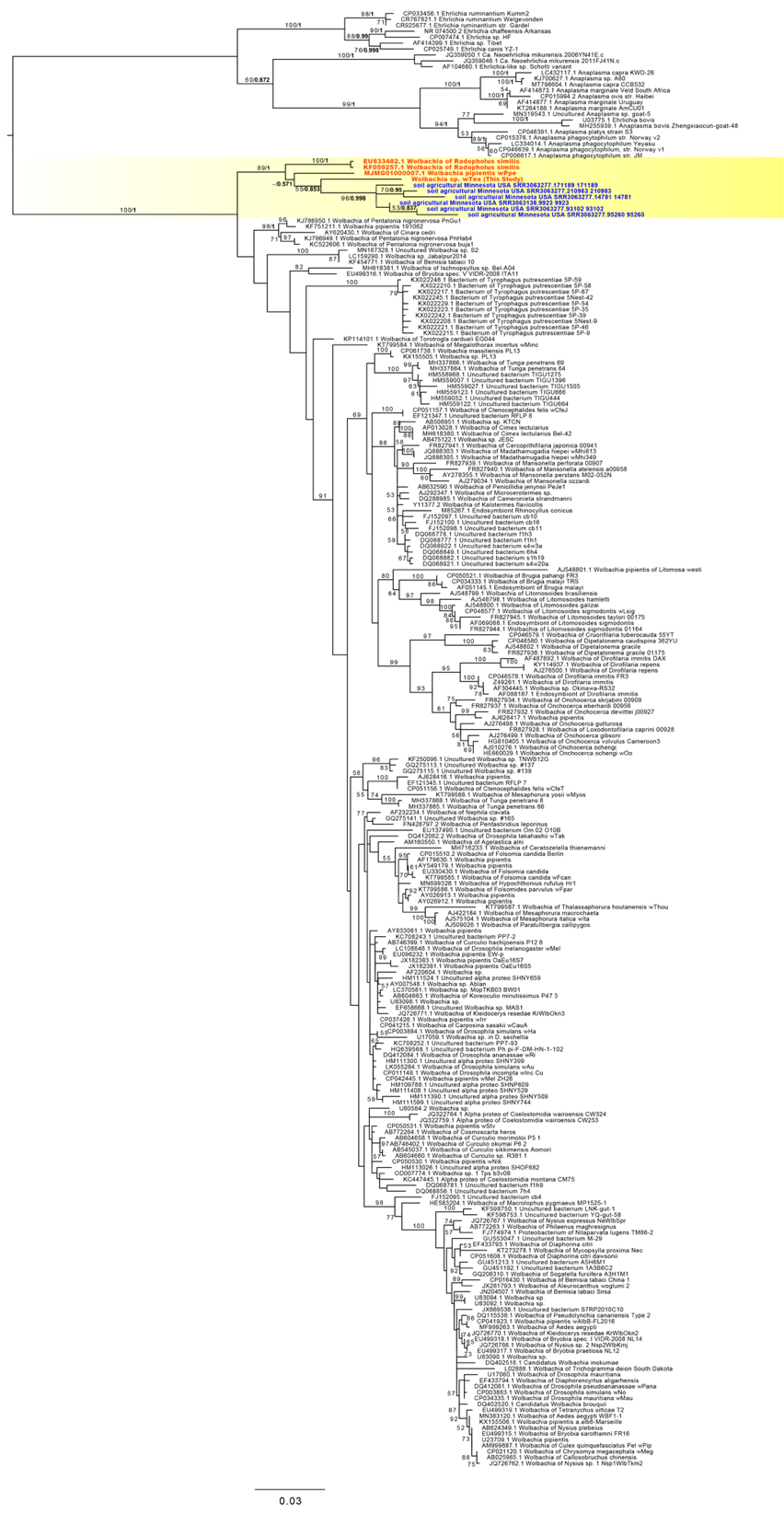
**

**Supplementary Figure 8**. Alignment of predicted dap operon genes showing synteny and sequence similarity among *asd2* (encoding aspartate-semialdehyde dehydrogenase 2) and *lysC* (encoding lysine-sensitive aspartokinase 3) among *Wolbachia* strains *w*Tex and *w*Ppe and *Candidatus* Midichloria mitochondrii. Top figure shows low-magnification alignment of predicted genes, with nucleotide identity plotted as a histogram above the sequence region. Bottom of figure shows predicted genes alongside nucleotide sequences denoted in grey for regions matching the consensus (majority) nucleotide or colored lines for nucleotide mismatches (red = A, purple = C, yellow = G, green = T) and showing a percent identity scale above each row. Flanking genes are *carA* (encoding carbamoyl-phosphate synthase small chain), *dapA* (encoding 4-hydroxy-tetrahydrodipicolinate synthase) and *lpxK* (encoding tetraacyldisaccharide 4'-kinase).

**
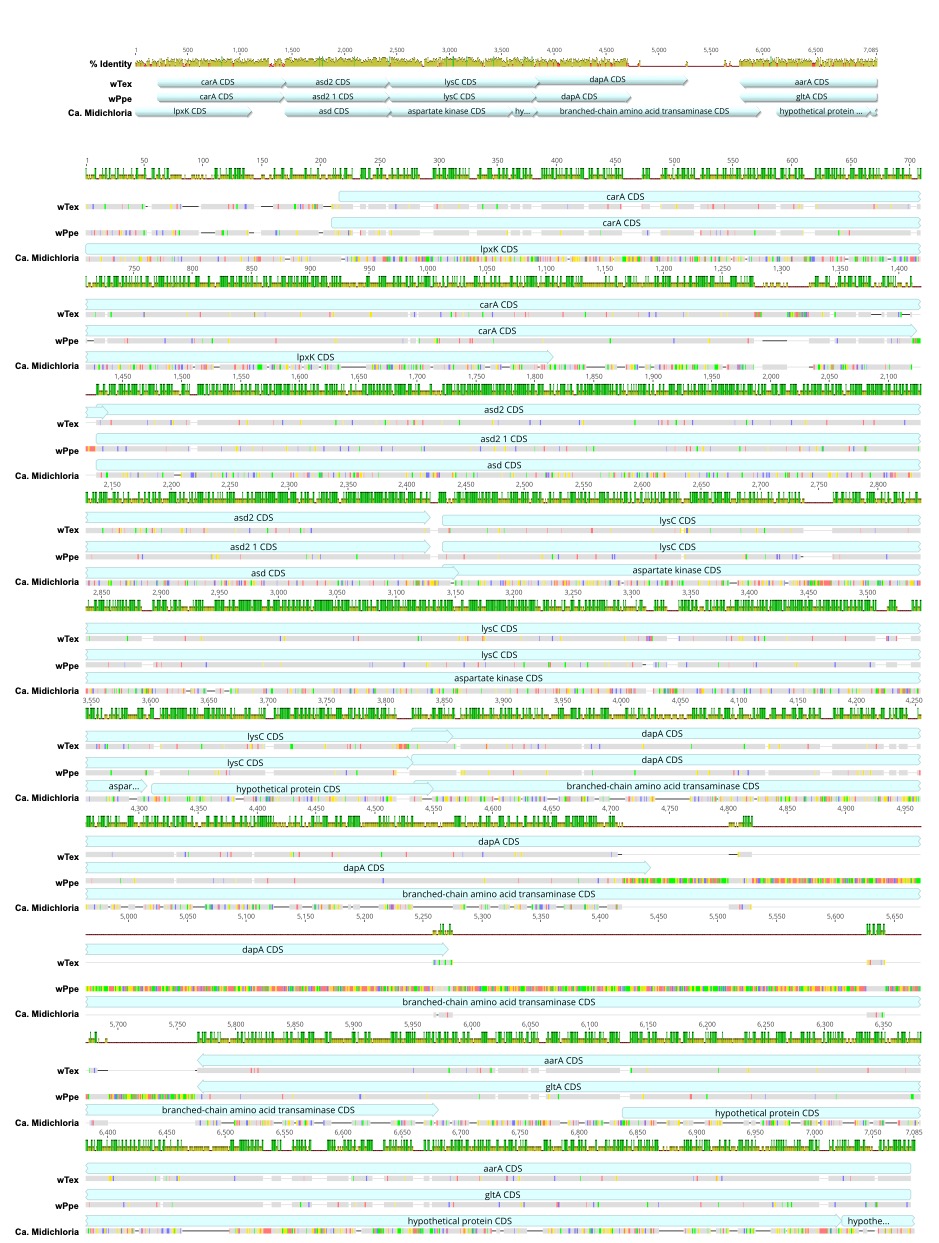
**

**Supplementary Figure 9**. Phylogeny of predicted Aspartate-semialdehyde dehydrogenase 2 (Asd2) homologs, based on 415 aligned amino acid positions, generated with maximum likelihood in RAxML with the GTR+Gamma model, showing bootstrap support >50% from 500 replicates on branches.

**
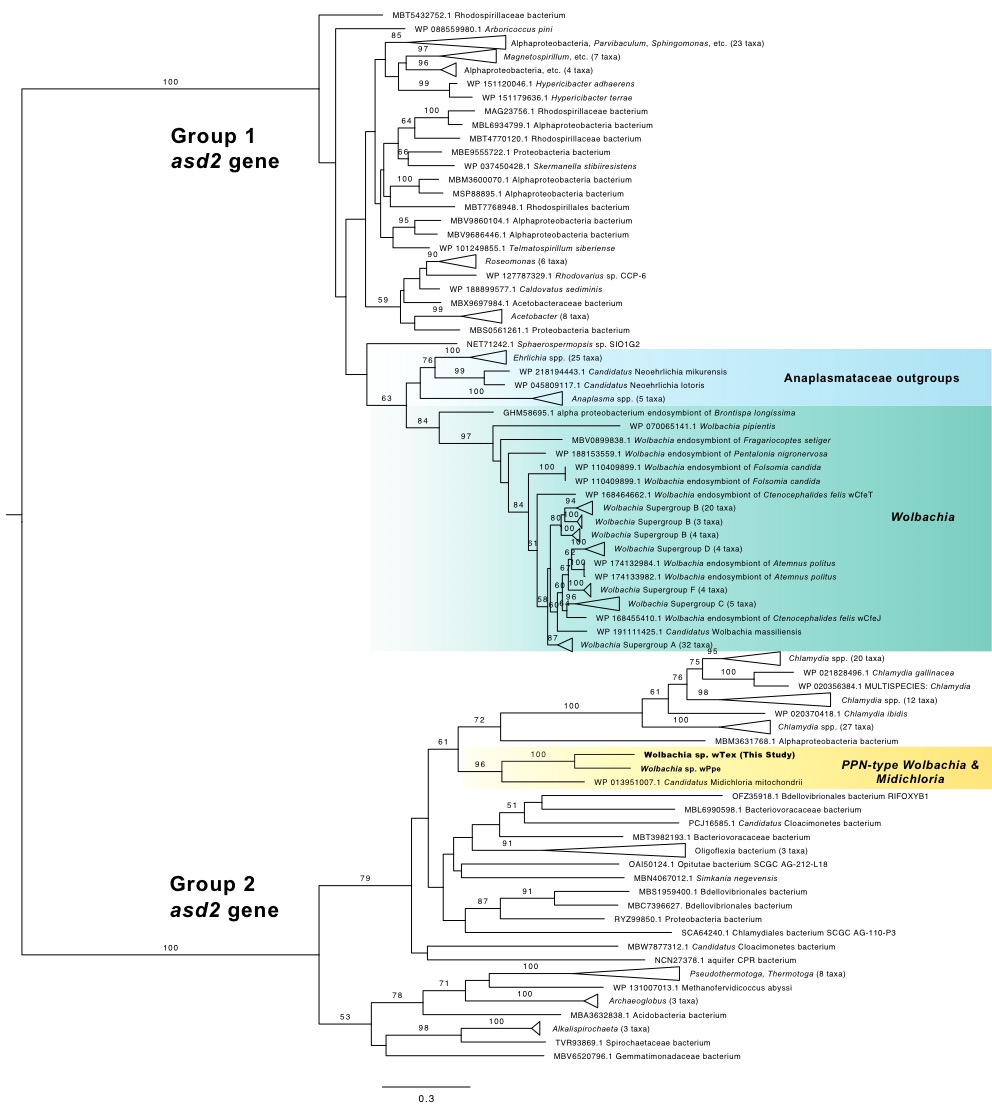
**

**Supplementary Figure 10**. Phylogeny of predicted Lysine-sensitive aspartokinase 3 (LysC) homologs, based on 502 aligned amino acid positions, generated with maximum likelihood in RAxML with the GTR+Gamma model, showing bootstrap support >50% from 500 replicates on branches.

**
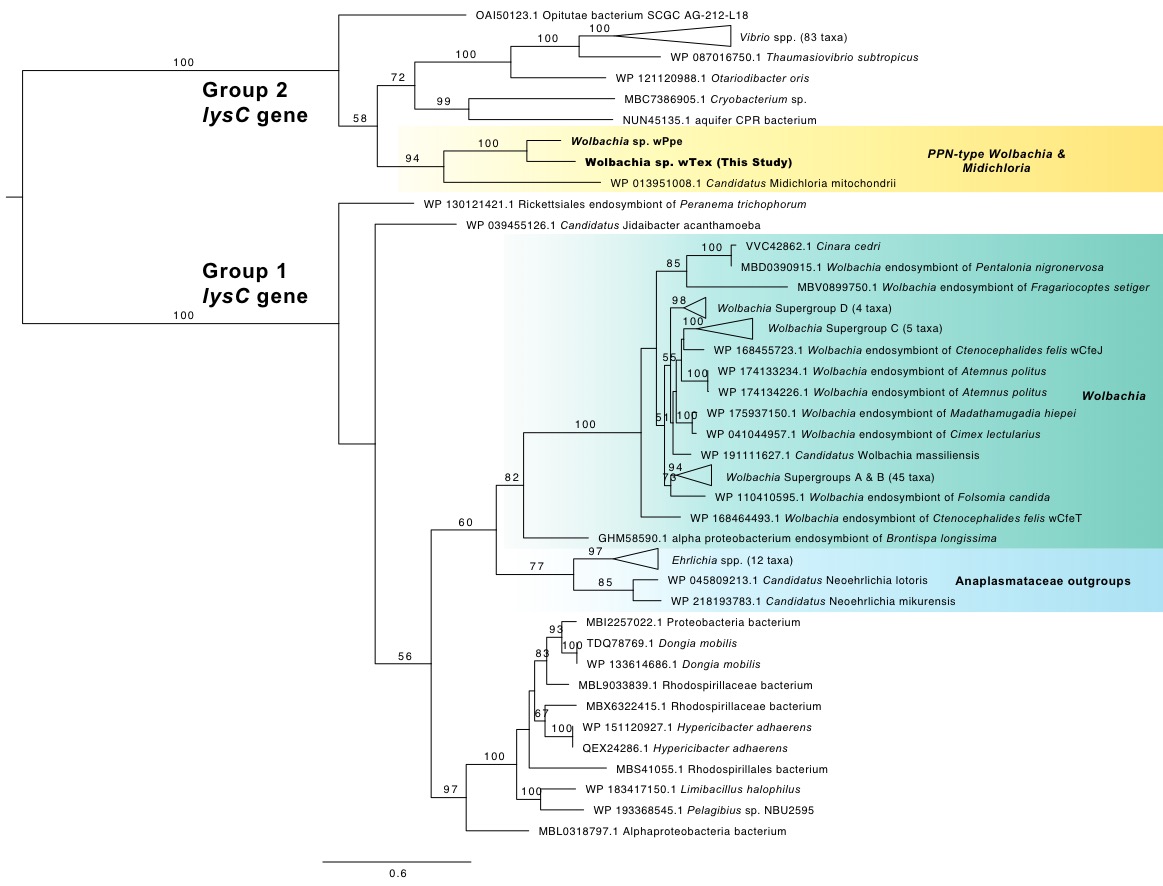
**

**Supplementary Table 3**: Significantly enriched gene ontology (GO) categories based on topGO analysis for the pangenome of *Wolbachia* *w*Tex compared to the pangenome of plant-parasitic nematode-associated *Wolbachia*, excluding genes shared between pangenomes.

| GO_ID | Term | Annot-  ated | Signif-  icant | Expected | P-value |
| --- | --- | --- | --- | --- | --- |
| **Biological Processes:** | | | | | |
| GO:0006313 | transposition, DNA-mediated | 39 | 20 | 6.4 | 1.5e-07 |
| GO:0009228 | thiamine biosynthetic process | 7 | 6 | 1.15 | 0.0001 |
| GO:0009229 | thiamine diphosphate biosynthetic process | 5 | 4 | 0.82 | 0.0030 |
| **Cellular Components:** | | | | | |
| GO:0016021 | integral component of membrane | 150 | 33 | 24.48 | 0.0078 |
| GO:0009376 | HslUV protease complex | 5 | 3 | 0.82 | 0.0325 |
| **Metabolic Functions:** | | | | | |
| GO:0004803 | transposase activity | 39 | 20 | 7.1 | 1.3e-06 |
| GO:0016740 | transferase activity | 167 | 37 | 30.41 | 0.00052 |
| GO:0003677 | DNA binding | 98 | 25 | 17.84 | 0.02740 |

**Supplementary Table 4**: Significantly enriched gene ontology (GO) categories based on topGO analysis for the pangenome of *Wolbachia* *w*Ppe compared to the pangenome of plant-parasitic nematode-associated *Wolbachia*, excluding genes shared between pangenomes.

| GO_ID | Term | Annot-  ated | Signif-  icant | Expected | P-value |
| --- | --- | --- | --- | --- | --- |
| **Biological Processes:** | | | | | |
| GO:0006298 | mismatch repair | 7 | 5 | 1.77 | 0.013 |
| GO:0006730 | one-carbon metabolic process | 7 | 5 | 1.77 | 0.016 |
| **Metabolic Functions:** | | | | | |
| GO:0003755 | peptidyl-prolyl cis-trans isomerase activity | 6 | 4 | 1.55 | 0.041 |

**Supplementary Table 5**: Significantly enriched gene ontology (GO) categories, based on topGO analysis, for the set of 126 genes in Figure 5 that includes shared core genes of plant-parasitic nematode-associated *Wolbachia* strains and these genes shared with at most two other strains of *Wolbachia*, compared with the ‘universe’ pangenome of early-branching strains *w*Tex, *w*Ppe, *w*Pni, *w*Fol, *w*CfeT, and *w*Chem.

| GO_ID | Term | Annot-  ated | Signif-  icant | Expected | P-value |
| --- | --- | --- | --- | --- | --- |
| **Biological Processes:** | | | | | |
| GO:0019877 | diaminopimelate biosynthetic process | 7 | 3 | 0.49 | 0.0096 |
| GO:0001522 | pseudouridine synthesis | 9 | 3 | 0.63 | 0.0208 |
| GO:0009089 | lysine biosynthetic process via diaminopimelate | 10 | 3 | 0.7 | 0.0282 |
| GO:0006094 | gluconeogenesis | 5 | 2 | 0.35 | 0.0427 |
| GO:0009423 | chorismate biosynthetic process | 5 | 2 | 0.35 | 0.0427 |
| **Metabolic Functions:** | | | | | |
| GO:0070006 | metalloaminopeptidase activity | 10 | 3 | 0.72 | 0.0299 |
| GO:0016829 | lyase activity | 73 | 12 | 5.25 | 0.0369 |
| GO:0004497 | monooxygenase activity | 11 | 3 | 0.79 | 0.0390 |
| GO:0005524 | ATP binding | 275 | 27 | 19.78 | 0.0440 |

**Supplementary Table 6**: Significantly enriched gene ontology (GO) categories based on topGO analysis for the pangenomes of plant-parasitic nematode-associated *Wolbachia* strains (*w*Tex and *w*Ppe) compared to the pangenome of all *Wolbachia*, including genes shared between pangenomes.

| GO_ID | Term | Annot-  ated | Signif-  icant | Expected | P-value |
| --- | --- | --- | --- | --- | --- |
| **Biological Processes:** | | | | | |
| GO:0006412 | translation | 233 | 84 | 34.27 | 2.9e-17 |
| GO:0015031 | protein transport | 90 | 26 | 13.24 | 0.0019 |
| GO:0006782 | protoporphyrinogen IX biosynthetic process | 7 | 5 | 1.03 | 0.0020 |
| GO:0009228 | thiamine biosynthetic process | 16 | 7 | 2.35 | 0.0049 |
| GO:0006235 | dTTP biosynthetic process | 10 | 5 | 1.47 | 0.0090 |
| GO:1902600 | proton transmembrane transport | 21 | 7 | 3.09 | 0.0112 |
| GO:0031564 | transcription antitermination | 7 | 4 | 1.03 | 0.0112 |
| GO:0009089 | lysine biosynthetic process via diaminopimelate | 15 | 6 | 2.21 | 0.0151 |
| GO:0007049 | cell cycle | 219 | 20 | 32.21 | 0.0190 |
| GO:0006744 | ubiquinone biosynthetic process | 29 | 9 | 4.27 | 0.0194 |
| GO:0006633 | fatty acid biosynthetic process | 20 | 7 | 2.94 | 0.0195 |
| GO:0046034 | ATP metabolic process | 82 | 17 | 12.06 | 0.0213 |
| GO:0019877 | diaminopimelate biosynthetic process | 12 | 5 | 1.76 | 0.0219 |
| GO:0009229 | thiamine diphosphate biosynthetic process | 12 | 5 | 1.76 | 0.0219 |
| GO:0045454 | cell redox homeostasis | 5 | 3 | 0.74 | 0.0251 |
| GO:0006189 | 'de novo' IMP biosynthetic process | 35 | 10 | 5.15 | 0.0252 |
| GO:0008360 | regulation of cell shape | 79 | 18 | 11.62 | 0.0348 |
| GO:0006351 | transcription, DNA-templated | 125 | 22 | 18.39 | 0.0429 |
| GO:0008652 | cellular amino acid biosynthetic process | 91 | 21 | 13.38 | 0.0437 |
| GO:0006231 | dTMP biosynthetic process | 6 | 3 | 0.88 | 0.0448 |
| GO:0006094 | gluconeogenesis | 6 | 3 | 0.88 | 0.0448 |
| GO:0009252 | peptidoglycan biosynthetic process | 69 | 15 | 10.15 | 0.0454 |
| GO:0032259 | methylation | 66 | 14 | 9.71 | 0.0493 |

**Supplementary Table 7**: Significantly enriched gene ontology (GO) categories based on topGO analysis for the core shared genes from plant-parasitic nematode-associated *Wolbachia* strains (*w*Tex and *w*Ppe) compared to the pangenome of all *Wolbachia*, including genes shared between pangenomes.

| GO_ID | Term | Annot-  ated | Signif-  icant | Expected | P-value |
| --- | --- | --- | --- | --- | --- |
| **Biological Processes:** | | | | | |
| GO:0006412 | translation | 233 | 62 | 20.01 | 1.9e-18 |
| GO:0031564 | transcription antitermination | 7 | 4 | 0.6 | 0.0015 |
| GO:0006782 | protoporphyrinogen IX biosynthetic process | 7 | 4 | 0.6 | 0.0055 |
| GO:0009089 | lysine biosynthetic process via diaminopimelate | 15 | 5 | 1.29 | 0.0066 |
| GO:0046034 | ATP metabolic process | 82 | 15 | 7.04 | 0.0071 |
| GO:0006744 | ubiquinone biosynthetic process | 29 | 7 | 2.49 | 0.0096 |
| GO:0006094 | gluconeogenesis | 6 | 3 | 0.52 | 0.0103 |
| GO:0006364 | rRNA processing | 33 | 8 | 2.83 | 0.0134 |
| GO:0019877 | diaminopimelate biosynthetic process | 12 | 4 | 1.03 | 0.0152 |
| GO:0006633 | fatty acid biosynthetic process | 20 | 5 | 1.72 | 0.0239 |
| GO:0006189 | 'de novo' IMP biosynthetic process | 35 | 7 | 3.01 | 0.0266 |
| GO:0055114 | oxidation-reduction process | 135 | 19 | 11.59 | 0.0277 |
| GO:0015031 | protein transport | 90 | 17 | 7.73 | 0.0287 |
| GO:0006235 | dTTP biosynthetic process | 10 | 3 | 0.86 | 0.0478 |

**Supplementary Table 8**: Significantly enriched gene ontology (GO) categories based on topGO analysis for the pangenomes of plant-parasitic nematode-associated *Wolbachia* strains (*w*Tex and *w*Ppe) compared to the pangenome of all *Wolbachia*, excluding genes shared between pangenomes.

| GO_ID | Term | Annot-  ated | Signif-  icant | Expected | P-value |
| --- | --- | --- | --- | --- | --- |
| **Biological Processes:** | | | | | |
| GO:0009228 | thiamine biosynthetic process | 16 | 3 | 0.35 | 0.0047 |
| GO:0008652 | cellular amino acid biosynthetic process | 91 | 7 | 2.01 | 0.0063 |
| GO:0006275 | regulation of DNA replication | 19 | 3 | 0.42 | 0.0078 |
| GO:0006270 | DNA replication initiation | 21 | 3 | 0.46 | 0.0103 |
| GO:0009236 | cobalamin biosynthetic process | 10 | 2 | 0.22 | 0.0193 |
| GO:0009229 | thiamine diphosphate biosynthetic process | 12 | 2 | 0.26 | 0.0275 |
| GO:0006730 | one-carbon metabolic process | 21 | 3 | 0.46 | 0.0411 |

**Supplementary Figure 11**: Alignment-wide dN/dS between *Wolbachia* strains *w*Tex and *w*Ppe based on dN/dS calculations for the complete ortholog alignment generated in Roary, for overlapping 1200 bp blocks, with gene ontology (GO) enrichment based on topGO analysis results for genes in top and bottom 10% and 10-25% of dN/dS values. Dots represent blocks in the aligned genomes. Pairwise dN/dS was calculated with KaKs Calculator. Dashed grey line shows the mean genome-wide dN/dS. Shaded areas show top and bottom portions of dN/dS values and associated GO terms that were significantly enriched at p-value <0.05. Full topGO results are shown in Supplementary Tables 9, 10, 11, and 12.

**
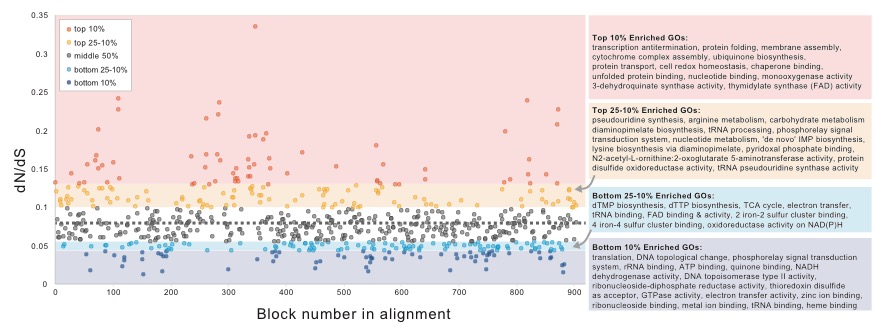
**

**Supplementary Table 9**: Significantly enriched gene ontology (GO) categories based on topGO analysis for the top 25% of dN/dS value genes from analysis of *Wolbachia* *w*Tex and *w*Ppe.

| GO_ID | Term | Annot-  ated | Signif-  icant | Expected | P-value |
| --- | --- | --- | --- | --- | --- |
| **Biological Processes:** | | | | | |
| GO:0031564 | transcription antitermination | 7 | 3 | 0.16 | 0.00038 |
| GO:0017004 | cytochrome complex assembly | 22 | 4 | 0.5 | 0.00135 |
| GO:0006744 | ubiquinone biosynthetic process | 29 | 4 | 0.66 | 0.00389 |
| GO:0001522 | pseudouridine synthesis | 27 | 4 | 0.62 | 0.00507 |
| GO:0006525 | arginine metabolic process | 26 | 4 | 0.59 | 0.00970 |
| GO:0006457 | protein folding | 74 | 7 | 1.69 | 0.01040 |
| GO:0005975 | carbohydrate metabolic process | 201 | 9 | 4.59 | 0.02163 |
| GO:0071709 | membrane assembly | 9 | 2 | 0.21 | 0.02262 |
| GO:0019877 | diaminopimelate biosynthetic process | 12 | 2 | 0.27 | 0.02931 |
| GO:0008033 | tRNA processing | 68 | 4 | 1.55 | 0.03359 |
| GO:0000160 | phosphorelay signal transduction system | 13 | 2 | 0.3 | 0.03413 |
| GO:0009117 | nucleotide metabolic process | 262 | 11 | 5.98 | 0.04293 |
| GO:0006189 | 'de novo' IMP biosynthetic process | 35 | 3 | 0.8 | 0.04449 |
| GO:0009089 | lysine biosynthetic process via diaminopimelate | 15 | 2 | 0.34 | 0.04462 |
| **Metabolic Functions:** | | | | | |
| GO:0030170 | pyridoxal phosphate binding | 37 | 4 | 0.86 | 0.010 |
| GO:0003992 | N2-acetyl-L-ornithine:2-oxoglutarate 5-aminotransferase activity | 7 | 2 | 0.16 | 0.010 |
| GO:0004497 | monooxygenase activity | 22 | 3 | 0.51 | 0.014 |
| GO:0015035 | protein disulfide oxidoreductase activity | 8 | 2 | 0.19 | 0.014 |
| GO:0000166 | nucleotide binding | 1110 | 31 | 25.76 | 0.014 |
| GO:0051087 | chaperone binding | 11 | 2 | 0.26 | 0.026 |
| GO:0106029 | tRNA pseudouridine synthase activity | 14 | 2 | 0.32 | 0.040 |

**Supplementary Table 10**: Significantly enriched gene ontology (GO) categories based on topGO analysis for the bottom 25% of dN/dS value genes from analysis of *Wolbachia* *w*Tex and *w*Ppe.

| GO_ID | Term | Annot-  ated | Signif-  icant | Expected | P-value |
| --- | --- | --- | --- | --- | --- |
| **Biological Processes:** | | | | | |
| GO:0006412 | translation | 233 | 33 | 6.95 | 4.7e-14 |
| GO:0006231 | dTMP biosynthetic process | 6 | 2 | 0.18 | 0.012 |
| GO:0006265 | DNA topological change | 17 | 3 | 0.51 | 0.013 |
| GO:0006235 | dTTP biosynthetic process | 10 | 2 | 0.3 | 0.034 |
| GO:0006099 | tricarboxylic acid cycle | 64 | 5 | 1.91 | 0.041 |
| **Cellular Components:** | | | | | |
| GO:0005840 | ribosome | 115 | 24 | 4.49 | 8.9e-10 |
| GO:0015934 | large ribosomal subunit | 10 | 4 | 0.39 | 0.00038 |
| **Metabolic Functions:** | | | | | |
| GO:0003735 | structural constituent of ribosome | 109 | 23 | 3.36 | 7.2e-14 |
| GO:0019843 | rRNA binding | 99 | 15 | 3.05 | 1.0e-06 |
| GO:0046872 | metal ion binding | 734 | 35 | 22.61 | 3.7e-05 |
| GO:0009055 | electron transfer activity | 59 | 9 | 1.82 | 0.00031 |
| GO:0000049 | tRNA binding | 58 | 7 | 1.79 | 0.00183 |
| GO:0050660 | flavin adenine dinucleotide binding | 69 | 7 | 2.13 | 0.00688 |
| GO:0008137 | NADH dehydrogenase (ubiquinone) activity | 27 | 4 | 0.83 | 0.00872 |
| GO:0005524 | ATP binding | 803 | 36 | 24.73 | 0.00995 |
| GO:0050797 | thymidylate synthase (FAD) activity | 6 | 2 | 0.18 | 0.01303 |
| GO:0051537 | 2 iron, 2 sulfur cluster binding | 36 | 4 | 1.11 | 0.02367 |
| GO:0003924 | GTPase activity | 73 | 6 | 2.25 | 0.02409 |
| GO:0051539 | 4 iron, 4 sulfur cluster binding | 75 | 6 | 2.31 | 0.02712 |
| GO:0048038 | quinone binding | 39 | 4 | 1.2 | 0.03080 |
| GO:0020037 | heme binding | 25 | 3 | 0.77 | 0.04010 |
| GO:0016651 | oxidoreductase activity, acting on NAD(P)H | 57 | 7 | 1.76 | 0.04120 |
| GO:0003918 | DNA topoisomerase type II (double strand cut, ATP-hydrolyzing) activity | 11 | 2 | 0.34 | 0.04319 |

**Supplementary Table 11**: Significantly enriched gene ontology (GO) categories based on topGO analysis for the top 10% of dN/dS value genes from analysis of *Wolbachia* *w*Tex and *w*Ppe.

| GO_ID | Term | Annot-  ated | Signif-  icant | Expected | P-value |
| --- | --- | --- | --- | --- | --- |
| **Biological Processes:** | | | | | |
| GO:0031564 | transcription antitermination | 7 | 2 | 0.06 | 0.0015 |
| GO:0006457 | protein folding | 74 | 5 | 0.63 | 0.0029 |
| GO:0071709 | membrane assembly | 9 | 2 | 0.08 | 0.0083 |
| GO:0017004 | cytochrome complex assembly | 22 | 2 | 0.19 | 0.0148 |
| GO:0006744 | ubiquinone biosynthetic process | 29 | 2 | 0.25 | 0.0250 |
| GO:0015031 | protein transport | 90 | 3 | 0.77 | 0.0405 |
| GO:0045454 | cell redox homeostasis | 5 | 1 | 0.04 | 0.0421 |
| **Metabolic Functions:** | | | | | |
| GO:0051087 | chaperone binding | 11 | 2 | 0.08 | 0.0030 |
| GO:0051082 | unfolded protein binding | 43 | 3 | 0.33 | 0.0040 |
| GO:0000166 | nucleotide binding | 1110 | 12 | 8.43 | 0.0112 |
| GO:0004497 | monooxygenase activity | 22 | 2 | 0.17 | 0.0118 |
| GO:0003856 | 3-dehydroquinate synthase activity | 5 | 1 | 0.04 | 0.0374 |
| GO:0050797 | thymidylate synthase (FAD) activity | 6 | 1 | 0.05 | 0.0447 |

**Supplementary Table 12**: Significantly enriched gene ontology (GO) categories based on topGO analysis for the bottom 10% of dN/dS value genes from analysis of *Wolbachia* *w*Tex and *w*Ppe.

| GO_ID | Term | Annot-  ated | Signif-  icant | Expected | P-value |
| --- | --- | --- | --- | --- | --- |
| **Biological Processes:** | | | | | |
| GO:0006412 | translation | 233 | 11 | 2.72 | 7.7e-05 |
| GO:0006265 | DNA topological change | 17 | 3 | 0.2 | 0.0009 |
| GO:0000160 | phosphorelay signal transduction system | 13 | 2 | 0.15 | 0.0096 |
| **Cellular Components:** | | | | | |
| GO:0015934 | large ribosomal subunit | 10 | 3 | 0.16 | 0.00043 |
| GO:0005840 | ribosome | 115 | 10 | 1.87 | 0.00145 |
| GO:0070469 | respirasome | 19 | 2 | 0.31 | 0.03677 |
| **Metabolic Functions:** | | | | | |
| GO:0003735 | structural constituent of ribosome | 109 | 9 | 1.35 | 5.8e-06 |
| GO:0019843 | rRNA binding | 99 | 7 | 1.22 | 0.00019 |
| GO:0005524 | ATP binding | 803 | 20 | 9.93 | 0.00097 |
| GO:0048038 | quinone binding | 39 | 4 | 0.48 | 0.00125 |
| GO:0008137 | NADH dehydrogenase (ubiquinone) activity | 27 | 3 | 0.33 | 0.00425 |
| GO:0003918 | DNA topoisomerase type II (double strand cut, ATP-hydrolyzing) activity | 11 | 2 | 0.14 | 0.00769 |
| GO:0004748 | ribonucleoside-diphosphate reductase activity, thioredoxin disulfide as acceptor | 12 | 2 | 0.15 | 0.00916 |
| GO:0003924 | GTPase activity | 73 | 4 | 0.9 | 0.01215 |
| GO:0009055 | electron transfer activity | 59 | 4 | 0.73 | 0.01596 |
| GO:0008270 | zinc ion binding | 128 | 5 | 1.58 | 0.02017 |
| GO:0032549 | ribonucleoside binding | 145 | 5 | 1.79 | 0.02359 |
| GO:0046872 | metal ion binding | 734 | 15 | 9.08 | 0.02591 |
| GO:0000049 | tRNA binding | 58 | 3 | 0.72 | 0.03424 |
| GO:0020037 | heme binding | 25 | 2 | 0.31 | 0.03757 |

**Supplementary Table 13**: Significantly enriched gene ontology (GO) categories based on topGO analysis for the bottom 10% of dN/dS value genes from analysis of *Wolbachia* *w*Ppe and *w*Pni.

| GO_ID | Term | Annot-  ated | Signif-  icant | Expected | P-value |
| --- | --- | --- | --- | --- | --- |
| **Biological Processes:** | | | | | |
| GO:0006265 | DNA topological change | 17 | 2 | 0.12 | 0.0065 |
| GO:0006526 | arginine biosynthetic process | 18 | 2 | 0.13 | 0.0072 |
| GO:0006352 | DNA-templated transcription, initiation | 31 | 2 | 0.23 | 0.0208 |
| GO:0043953 | protein transport by the Tat complex | 6 | 1 | 0.04 | 0.0428 |
| GO:0044205 | 'de novo' UMP biosynthetic process | 47 | 2 | 0.34 | 0.0450 |
| GO:0051603 | proteolysis involved in cellular protein catabolic process | 7 | 1 | 0.05 | 0.0498 |
| **Cellular Components:** | | | | | |
| GO:0009376 | HslUV protease complex | 9 | 2 | 0.09 | 0.0034 |
| GO:0070469 | respirasome | 19 | 2 | 0.19 | 0.0151 |
| **Metabolic Functions:** | | | | | |
| GO:0003899 | DNA-directed 5'-3' RNA polymerase activity | 16 | 3 | 0.14 | 0.00029 |
| GO:0003924 | GTPase activity | 73 | 4 | 0.62 | 0.00313 |
| GO:0003918 | DNA topoisomerase type II (double strand cut, ATP-hydrolyzing) activity | 11 | 2 | 0.09 | 0.00366 |
| GO:0048038 | quinone binding | 39 | 3 | 0.33 | 0.00415 |
| GO:0005524 | ATP binding | 803 | 14 | 6.79 | 0.00433 |
| GO:0004748 | ribonucleoside-diphosphate reductase activity, thioredoxin disulfide as acceptor | 12 | 2 | 0.1 | 0.00436 |
| GO:0005525 | GTP binding | 143 | 5 | 1.21 | 0.00659 |
| GO:0004088 | carbamoyl-phosphate synthase (glutamine-hydrolyzing) activity | 16 | 2 | 0.14 | 0.00777 |
| GO:0032549 | ribonucleoside binding | 145 | 6 | 1.23 | 0.01517 |
| GO:0016987 | sigma factor activity | 24 | 2 | 0.2 | 0.01712 |
| GO:0008137 | NADH dehydrogenase (ubiquinone) activity | 27 | 2 | 0.23 | 0.02143 |
| GO:0003938 | IMP dehydrogenase activity | 5 | 1 | 0.04 | 0.04161 |
| GO:0003729 | mRNA binding | 6 | 1 | 0.05 | 0.04972 |

**Supplementary Table 14**: Significantly enriched gene ontology (GO) categories based on topGO analysis for the top 10% of dN/dS value genes from analysis of *Wolbachia* *w*Ppe and *w*Pni.

| GO_ID | Term | Annot-  ated | Signif-  icant | Expected | P-value |
| --- | --- | --- | --- | --- | --- |
| **Biological Processes:** | | | | | |
| GO:0017004 | cytochrome complex assembly | 22 | 2 | 0.12 | 0.0061 |
| GO:0008360 | regulation of cell shape | 79 | 3 | 0.43 | 0.0084 |
| GO:0006426 | glycyl-tRNA aminoacylation | 7 | 1 | 0.04 | 0.0375 |
| GO:0006048 | UDP-N-acetylglucosamine biosynthetic process | 8 | 1 | 0.04 | 0.0428 |
| GO:0019684 | photosynthesis, light reaction | 8 | 1 | 0.04 | 0.0428 |
| GO:0051301 | cell division | 147 | 3 | 0.8 | 0.0437 |
| GO:0071709 | membrane assembly | 9 | 1 | 0.05 | 0.0480 |
| **Cellular Components:** | | | | | |
| GO:0016021 | integral component of membrane | 762 | 13 | 7.73 | 0.024 |
| **Metabolic Functions:** | | | | | |
| GO:0008765 | UDP-N-acetylmuramoylalanyl-D-glutamate-2,6-diaminopimelate ligase activity | 6 | 1 | 0.03 | 0.0334 |
| GO:0004820 | glycine-tRNA ligase activity | 7 | 1 | 0.04 | 0.0388 |
| GO:0015035 | protein disulfide oxidoreductase activity | 8 | 1 | 0.05 | 0.0443 |
| GO:0003977 | UDP-N-acetylglucosamine diphosphorylase activity | 9 | 1 | 0.05 | 0.0497 |
| GO:0019134 | glucosamine-1-phosphate N-acetyltransferase activity | 9 | 1 | 0.05 | 0.0497 |

**Supplementary Table 15**: Significantly enriched gene ontology (GO) categories based on topGO analysis for the bottom 10% of dN/dS value genes from analysis of *Wolbachia* *w*Tex and *w*Pni.

| GO_ID | Term | Annot-  ated | Signif-  icant | Expected | P-value |
| --- | --- | --- | --- | --- | --- |
| **Biological Processes:** | | | | | |
| GO:0006265 | DNA topological change | 17 | 2 | 0.07 | 0.0024 |
| GO:0006412 | translation | 233 | 4 | 1.03 | 0.0213 |
| GO:0006261 | DNA-dependent DNA replication | 56 | 2 | 0.25 | 0.0245 |
| GO:0006782 | protoporphyrinogen IX biosynthetic process | 7 | 1 | 0.03 | 0.0305 |
| **Metabolic Functions:** | | | | | |
| GO:0048038 | quinone binding | 39 | 3 | 0.19 | 0.00077 |
| GO:0003918 | DNA topoisomerase type II (double strand cut, ATP-hydrolyzing) activity | 11 | 2 | 0.05 | 0.00117 |
| GO:0004748 | ribonucleoside-diphosphate reductase activity, thioredoxin disulfide as acceptor | 12 | 2 | 0.06 | 0.00139 |
| GO:0000049 | tRNA binding | 58 | 3 | 0.28 | 0.00246 |
| GO:0003924 | GTPase activity | 73 | 3 | 0.35 | 0.00473 |
| GO:0008137 | NADH dehydrogenase (ubiquinone) activity | 27 | 2 | 0.13 | 0.00710 |
| GO:0032549 | ribonucleoside binding | 145 | 4 | 0.69 | 0.00849 |
| GO:0019843 | rRNA binding | 99 | 3 | 0.47 | 0.01100 |
| GO:0003735 | structural constituent of ribosome | 109 | 3 | 0.52 | 0.01428 |
| GO:0051287 | NAD binding | 43 | 2 | 0.21 | 0.01744 |
| GO:0005524 | ATP binding | 803 | 8 | 3.83 | 0.02653 |
| GO:0005525 | GTP binding | 143 | 3 | 0.68 | 0.02923 |

**Supplementary Table 16**: Significantly enriched gene ontology (GO) categories based on topGO analysis for the top 10% of dN/dS value genes from analysis of *Wolbachia* *w*Tex and *w*Pni.

| GO_ID | Term | Annot-  ated | Signif-  icant | Expected | P-value |
| --- | --- | --- | --- | --- | --- |
| **Biological Processes:** | | | | | |
| GO:1902600 | proton transmembrane transport | 21 | 2 | 0.08 | 0.0217 |
| **Metabolic Functions:** | | | | | |
| GO:0008962 | phosphatidylglycerophosphatase activity | 5 | 1 | 0.02 | 0.0215 |
| GO:0004618 | phosphoglycerate kinase activity | 6 | 1 | 0.03 | 0.0258 |

**Supplementary Table 17**: Significantly enriched gene ontology (GO) categories based on topGO analysis for the bottom 10% of dN/dS value genes from analysis of *Wolbachia* *w*Pni and *w*Fol.

| GO_ID | Term | Annot-  ated | Signif-  icant | Expected | P-value |
| --- | --- | --- | --- | --- | --- |
| **Biological Processes:** | | | | | |
| GO:0006412 | translation | 233 | 10 | 2.48 | 0.011 |
| GO:0006265 | DNA topological change | 17 | 2 | 0.18 | 0.014 |
| GO:0006099 | tricarboxylic acid cycle | 64 | 3 | 0.68 | 0.030 |
| **Cellular Components:** | | | | | |
| GO:0070469 | respirasome | 19 | 3 | 0.24 | 0.0015 |
| GO:0015934 | large ribosomal subunit | 10 | 2 | 0.13 | 0.0065 |
| **Metabolic Functions:** | | | | | |
| GO:0005524 | ATP binding | 803 | 22 | 9.93 | 0.00011 |
| GO:0004129 | cytochrome-c oxidase activity | 15 | 3 | 0.19 | 0.00073 |
| GO:0004775 | succinate-CoA ligase (ADP-forming) activity | 6 | 2 | 0.07 | 0.00218 |
| GO:0003918 | DNA topoisomerase type II (double strand cut, ATP-hydrolyzing) activity | 11 | 2 | 0.14 | 0.00769 |
| GO:0004748 | ribonucleoside-diphosphate reductase activity, thioredoxin disulfide as acceptor | 12 | 2 | 0.15 | 0.00916 |
| GO:0048038 | quinone binding | 39 | 3 | 0.48 | 0.01195 |
| GO:0003924 | GTPase activity | 73 | 4 | 0.9 | 0.01215 |
| GO:0003899 | DNA-directed 5'-3' RNA polymerase activity | 16 | 2 | 0.2 | 0.01613 |
| GO:0009378 | four-way junction helicase activity | 16 | 2 | 0.2 | 0.01613 |
| GO:0032549 | ribonucleoside binding | 145 | 6 | 1.79 | 0.02315 |
| GO:0003746 | translation elongation factor activity | 53 | 3 | 0.66 | 0.02714 |
| GO:0005525 | GTP binding | 143 | 5 | 1.77 | 0.03079 |
| GO:0008137 | NADH dehydrogenase (ubiquinone) activity | 27 | 2 | 0.33 | 0.04327 |
| GO:0003735 | structural constituent of ribosome | 109 | 4 | 1.35 | 0.04483 |

**Supplementary Table 18**: Significantly enriched gene ontology (GO) categories based on topGO analysis for the top 10% of dN/dS value genes from analysis of *Wolbachia* *w*Pni and *w*Fol.

| GO_ID | Term | Annot-  ated | Signif-  icant | Expected | P-value |
| --- | --- | --- | --- | --- | --- |
| **Biological Processes:** | | | | | |
| GO:0042274 | ribosomal small subunit biogenesis | 15 | 2 | 0.08 | 0.0028 |
| GO:0006364 | rRNA processing | 33 | 3 | 0.18 | 0.0089 |
| GO:0046677 | response to antibiotic | 31 | 2 | 0.17 | 0.0120 |
| GO:0031167 | rRNA methylation | 5 | 1 | 0.03 | 0.0270 |
| GO:0019632 | shikimate metabolic process | 5 | 1 | 0.03 | 0.0270 |
| **Metabolic Functions:** | | | | | |
| GO:0008840 | 4-hydroxy-tetrahydrodipicolinate synthase activity | 5 | 1 | 0.03 | 0.0268 |
| GO:0004765 | shikimate kinase activity | 5 | 1 | 0.03 | 0.0268 |
| GO:0043022 | ribosome binding | 6 | 1 | 0.03 | 0.0321 |
| GO:0008168 | methyltransferase activity | 98 | 3 | 0.53 | 0.0332 |
| GO:0000179 | rRNA (adenine-N6,N6-)-dimethyltransferase activity | 7 | 1 | 0.04 | 0.0374 |
| GO:0052908 | 16S rRNA (adenine(1518)-N(6)/adenine(1519)-N(6))-dimethyltransferase activity | 7 | 1 | 0.04 | 0.0374 |
| GO:0001671 | ATPase activator activity | 7 | 1 | 0.04 | 0.0374 |

**Supplementary Table 19**: Significantly enriched gene ontology (GO) categories based on topGO analysis for the bottom 10% of dN/dS value genes from analysis of *Wolbachia* *w*Fol and *w*CfeT.

| GO_ID | Term | Annot-  ated | Signif-  icant | Expected | P-value |
| --- | --- | --- | --- | --- | --- |
| **Biological Processes:** | | | | | |
| GO:0006265 | DNA topological change | 17 | 3 | 0.22 | 0.00116 |
| GO:0045454 | cell redox homeostasis | 5 | 2 | 0.06 | 0.00154 |
| GO:0006099 | tricarboxylic acid cycle | 64 | 4 | 0.81 | 0.00837 |
| **Cellular Components:** | | | | | |
| GO:0070469 | respirasome | 19 | 3 | 0.3 | 0.0029 |
| GO:0015934 | large ribosomal subunit | 10 | 2 | 0.16 | 0.0100 |
| GO:0015935 | small ribosomal subunit | 14 | 2 | 0.22 | 0.0194 |
| **Metabolic Functions:** | | | | | |
| GO:0005524 | ATP binding | 803 | 28 | 11.84 | 3e-06 |
| GO:0046872 | metal ion binding | 734 | 21 | 10.83 | 0.00044 |
| GO:0048038 | quinone binding | 39 | 4 | 0.58 | 0.00241 |
| GO:0004775 | succinate-CoA ligase (ADP-forming) activity | 6 | 2 | 0.09 | 0.00310 |
| GO:0008137 | NADH dehydrogenase (ubiquinone) activity | 27 | 3 | 0.4 | 0.00697 |
| GO:0000049 | tRNA binding | 58 | 4 | 0.86 | 0.01010 |
| GO:0003918 | DNA topoisomerase type II (double strand cut, ATP-hydrolyzing) activity | 11 | 2 | 0.16 | 0.01082 |
| GO:0004129 | cytochrome-c oxidase activity | 15 | 2 | 0.22 | 0.01989 |
| GO:0051539 | 4 iron, 4 sulfur cluster binding | 75 | 4 | 1.11 | 0.02403 |
| GO:0009055 | electron transfer activity | 59 | 5 | 0.87 | 0.02466 |
| GO:0051287 | NAD binding | 43 | 3 | 0.63 | 0.02488 |
| GO:0032549 | ribonucleoside binding | 145 | 4 | 2.14 | 0.02889 |

**Supplementary Table 20**: Significantly enriched gene ontology (GO) categories based on topGO analysis for the top 10% of dN/dS value genes from analysis of *Wolbachia* *w*Fol and *w*CfeT.

| GO_ID | Term | Annot-  ated | Signif-  icant | Expected | P-value |
| --- | --- | --- | --- | --- | --- |
| **Biological Processes:** | | | | | |
| GO:0006189 | 'de novo' IMP biosynthetic process | 35 | 2 | 0.21 | 0.018 |
| GO:0031167 | rRNA methylation | 5 | 1 | 0.03 | 0.029 |
| GO:0051205 | protein insertion into membrane | 7 | 1 | 0.04 | 0.041 |
| GO:0006426 | glycyl-tRNA aminoacylation | 7 | 1 | 0.04 | 0.041 |
| GO:0019684 | photosynthesis, light reaction | 8 | 1 | 0.05 | 0.047 |
| GO:0043165 | Gram-negative-bacterium-type cell outer membrane assembly | 8 | 1 | 0.05 | 0.047 |
| **Metabolic Functions:** | | | | | |
| GO:0015297 | antiporter activity | 27 | 2 | 0.16 | 0.0117 |
| GO:0030976 | thiamine pyrophosphate binding | 6 | 1 | 0.04 | 0.0359 |
| GO:0004820 | glycine-tRNA ligase activity | 7 | 1 | 0.04 | 0.0418 |
| GO:0000179 | rRNA (adenine-N6,N6-)-dimethyltransferase activity | 7 | 1 | 0.04 | 0.0418 |
| GO:0052908 | 16S rRNA (adenine(1518)-N(6)/adenine(1519)-N(6))-dimethyltransferase activity | 7 | 1 | 0.04 | 0.0418 |
| GO:0008324 | cation transmembrane transporter activity | 117 | 3 | 0.71 | 0.0453 |
| GO:0061712 | tRNA (N(6)-L-threonylcarbamoyladenosine(37)-C(2))-methylthiotransferase | 8 | 1 | 0.05 | 0.0476 |
| GO:0016726 | oxidoreductase activity, acting on CH or CH2 groups, NAD or NADP as acceptor | 8 | 1 | 0.05 | 0.0476 |
